# Dependency Between Protein–Protein Interactions and Protein Variability and Evolutionary Rates in Vertebrates: Observed Relationships and Stochastic Modeling

**DOI:** 10.1007/s00239-019-09899-z

**Published:** 2019-07-13

**Authors:** Xichun Wang, Sergio Branciamore, Grigoriy Gogoshin, Andrei S. Rodin

**Affiliations:** Department of Computational and Quantitative Medicine and Diabetes and Metabolism Research Institute, Beckman Research Institute of the City of Hope, 1500 East Duarte Road, Duarte, CA 91010-3000 USA

**Keywords:** Protein connectivity, Protein variability, Protein–protein interactions, PPI, Stochastic computer simulations, Protein evolutionary rates

## Abstract

Recent developments in sequencing and growth of bioinformatics resources provide us with vast depositories of protein network and single nucleotide polymorphism data. It allows us to re-examine, on a larger and more comprehensive scale, the relationship between protein–protein interactions and protein variability and evolutionary rates. This relationship has remained far from unambiguously resolved for quite a long time, reflecting shifting analysis approaches in the literature, and growing data availability. In this study, we utilized several public genomic databases to investigate this relationship in human, mouse, pig, chicken, and zebrafish. We observed strong non-linear relationship patterns (tending towards convex decreasing function shapes) between protein variability and the density of corresponding protein–protein interactions across all five species. To investigate further, we carried out stochastic simulations, modeling the interplay between protein connectivity and variability. Our results indicate that a simple negative linear correlation model, often suggested (or tacitly assumed) in the literature, as either a null or an alternative hypothesis, is not a good fit with the observed data. After considering different (but still relatively simple, and not overfitting) simulation models, we found that a convex decreasing protein variability–connectivity function (specifically, exponential decay) led to a much better fit with the real data. We conclude that simple correlation models might be inadequate for describing protein variability–connectivity interplay in vertebrates; they often tend towards false negatives (showing no more than marginal linear or rank correlation where there are in fact strong non-random patterns).

**Electronic supplementary material:**

The online version of this article (10.1007/s00239-019-09899-z) contains supplementary material, which is available to authorized users.

## Introduction

Protein–protein interactions (PPIs) play an important role in realizing specific functions in biological systems. One way to represent PPIs is via protein networks. In a protein network, a link between two proteins is a visualization of a dependency relationship. The latter can have many biological meanings, ranging from direct or indirect physical/chemical interactions to co-expression to ontological proximity (Asur et al. 2007). Protein networks have attractive mathematical properties—interpretability, compartmentalization, scalability, admixture of different types of proteins and dependencies, and scale-free properties coupled with the network sparseness (Nacher et al. 2009). However, translation “back” from the protein network abstraction into the actual mechanistic understanding of the biochemical machinery (and its regulation and evolutionary history, up to the genomic level) is not a trivial undertaking (Ghadie et al. 2017; Guo et al. 2014; Kirk et al. 2017a, b; Zhu et al. 2013). In this light, a relationship between the “connectivity” of a protein (i.e., the density of its immediate Markov neighborhood in the network, also correlated with its “centrality” in the network, and its network “hub,” as opposed to “periphery,” positioning) and its evolutionary qualities/parameters (such as intraspecific variability, duplicability, and interspecific variability) is of a special interest.

Establishing (and quantifying) this relationship has been a notable research goal over the last 10–15 years [see (Zhu et al. 2012) for a broad discussion]. Prachumwat and Li demonstrated an inverse relationship between high connectivity and gene duplicability (with older genes tending towards higher connectivity) in yeast (Prachumwat and Li 2006). However, later studies suggested direct proportionality in mammals (Liang and Li 2007), leading the authors to speculate that highly connected proteins require higher dosages, thus necessitating more gene duplication (bringing about, in turn, more functional diversification). In parallel, higher gene duplicability was hypothesized to be linked with increased protein complexity (via longer protein sequences and higher number of functional domains) (Yang et al. 2003).

Switching from duplicability to variability—early on Fraser et al. have demonstrated negative correlation between protein interactions and evolutionary rate (on the basis of yeast species and *C. elegans*) (Fraser et al. 2002, 2003). Jordan et al. argued that only very weak correlation (if any at all) could be found between protein evolutionary rate and protein connectivity in yeast species (Jordan et al. 2003). This inter-study discrepancy could be attributed to the differences between (and consequent unintended biases stemming from) the datasets used, and other analysis artifacts (Bloom and Adami 2003)—raising, of course, the question of the robustness of the general investigative approach in the first place (Plotkin and Fraser 2007). Perhaps even more importantly, protein evolutionary rates are determined and influenced by many other factors, and correlate (negatively or positively) with many other measurements (Alvarez-Ponce and Fares 2012; Alvarez-Ponce et al. 2017; Josephs et al. 2017; Liao et al. 2006; Mahler et al. 2017; Saeed and Deane 2006; Zhang and Yang 2015)—it has been suggested that weak-to-moderate negative correlation between protein connectivity and variability might not signify causation per se, but rather reflect strong correlation between protein connectivity/PPI and other factors influencing protein evolutionary rates (Koonin and Wolf 2006)—and, in any case, protein connectivity might be insignificant in comparison to some of these other factors. Specifically, expression levels and patterns (such as histological breadth) have been singled out as important factors (Drummond et al. 2005; Mahler et al. 2017; Pal et al. 2001), with protein connectivity/PPI’s independent contribution to the protein variability estimated to be comparatively negligible (Drummond et al. 2006; Yang and Gaut 2011) [but see (Plotkin and Fraser 2007; Alvarez-Ponce et al. 2017) for a somewhat different prospective]. Much of the above work has been carried out in yeast—however, it was recently demonstrated, in *H. sapiens*, and other species, that protein connectivity/centrality still impacts protein variability, independently of gene expression levels (Alvarez-Ponce et al. 2017; Josephs et al. 2017; Masalia et al. 2017), and that there is a marginal-to-strong negative correlation between protein connectivity/centrality and genetic divergence.

It should also be noted that there is a difference between the topological localization in the PPI network (e.g., “hub” vs. “periphery”) and protein connectivity—although it has been argued that the “hub” protein assignment might be a somewhat artificial notion (Batada et al. 2006, 2007), there is significant recent evidence that “hub” proteins are subjected to strong negative selection (Biswas et al. 2017; Kirk et al. 2017a; Pang et al. 2016). Finally, a distinction should also be made, when talking about protein evolutionary rates, between protein intraspecific variability, polymorphism, and interspecific variability (divergence). In conclusion, the general question of variability–connectivity correlation remains unresolved to a significant degree.

In our opinion, one of the hitherto ignored angles is the way “correlation” per se is defined and measured. In much of the above literature, single-nucleotide polymorphisms (SNPs) are counted in various species datasets available as public resources, and then, after invoking basic transformations (e.g., computing nonsynonymous/synonymous rate ratios), linear correlation coefficients (or their non-parametric, rank correlation, equivalents) between protein evolutionary rate and connectivity are derived. However, such simple relationships might be a poor fit in this particular situation. In general, it is possible, even likely, to mistake even a very pronounced pattern for the absence of correlation in biological systems if an overly simplistic model is used for the correlation analysis (Dietrich 1991); we believe that this might have been happening here, contributing to the aforementioned ambiguity. Therefore, in our approach, we aimed at examining the link between protein connectivity and variability as a complex and (possibly) non-linear dependency, on the distributional level. We also wanted to extend the analysis to as many proteins as practically feasible, taking advantage of the ever-growing public genome database resources, and to concentrate on the vertebrate species, to complement the existing analyses in yeast, *C. elegans*, and flowering plants.

After analyzing SNP data from five different species (human, mouse, pig, chicken, zebrafish) using different public genomic datasets, we confirm that there is a tendency towards negative dependency between protein connectivity and protein variability at the interspecific (ortholog) variability level (but not at the intraspecific variability level). However, this relationship is clearly non-linear; corresponding distributions exhibit a distinct shape largely invariant across the different species and databases. Using simulations, we show that incorporation of the non-linear (namely, exponential decay) variability–connectivity functions in the simulated evolutionary process results in the variability–connectivity relationship patterns that approximate the observed, real, protein variability–connectivity data sufficiently well; along the way, we propose a simple but mathematically rigorous way to stochastically model the interplay of protein connectivity and protein evolutionary rates.

## Materials and Methods

### Database Data (Protein Connectivity and Evolutionary Rates)

Our principal analyses were centered around the protein connectivity data for human, mouse, pig, chicken, and zebrafish assembled in the STRING database (Szklarczyk et al. 2017). In STRING database, each protein–protein connection is assigned a probability score (an estimate of whether the connection in question is biologically meaningful, specific, and reproducible). Only confirmed physical non-redundant connections with significant scores were considered. We started by selecting all connections for which the corresponding intraspecific protein evolutionary rate data were available, resulting in 15,903 human proteins, 12,937 mouse proteins, 1017 pig proteins, 804 chicken proteins, and 2124 zebrafish proteins. We continued by compiling all connections for which confirmed (Wolf and Koonin 2012) human ortholog (human/chimpanzee, specifically) and mouse ortholog (mouse/rat) data were available, resulting in 15,116 human proteins and 15,246 mouse proteins. For these, standard built-in Ensembl Genome Browser phylogenetic analysis scheme, encompassing maximum likelihood tree reconstruction and maximum likelihood dN (nonsynonymous substitution rate) and dS (synonymous substitution rate) estimation, was used to obtain dN, dS, and dN/dS values (Aken et al. 2016; Chen et al. 2010; Yang 1997).

Importantly, we used both intraspecific protein evolutionary rate data and interspecific protein evolutionary rate (divergence, or ortholog) data throughout the study. Both present biological interest; in addition, the former is more congruent with our simulation framework (as detailed below), while the latter is directly comparable with the majority of the results in the literature. We used total SNP counts, dN, dS, and dN/dS ratio to evaluate protein variability. The former are, again, more congruent with our simulation framework, while dN and dN/dS ratio arguably do the best job of quantifying selection pressures in sufficiently divergent sequences (unless the synonymous sites are under significant selection pressure, which is unlikely to be the case for the five species in this present study). We did not utilize intraspecific dN/dS data, because it is unclear whether dN/dS is at all meaningful in the context of segregating polymorphisms (Kryazhimskiy and Plotkin 2008).

These analyses were augmented with the separate analyses of the human protein connectivity data from the Reactome database (Fabregat et al. 2016). Protein–protein interactions in Reactome are further classified into four groups: “direct complex” (interactions between proteins present in the same complex), “indirect complex” (present in different subcomplexes of a complex), “reaction” (participating in a reaction but not present in the same complex), and “neighboring reaction” (participating in two consecutive reactions but not present in the same complex). The latter two categories are not, strictly speaking, physical PPI—nevertheless, we have included them in the (separate) analyses, for comparison/control purposes. After the data cleanup (removing self-connections, etc.), the human intraspecific variability Reactome dataset contained 1839 proteins in “direct complex” group, 2109 proteins in “indirect complex” group, 2975 proteins in “reaction” group, and 3187 proteins in “neighboring reaction” group. Human/chimpanzee ortholog Reactome dataset contained 1729 proteins in “direct complex” group, 2001 proteins in “indirect complex” group, 2824 proteins in “reaction” group, and 3072 proteins in “neighboring reaction” group. Finally, we have also carried out the separate analyses for human proteins from Agile Protein Interactomes DataServer (APID) (Alonso-Lopez et al. 2016). Only confirmed physical interactions were included. These amounted to 15,651 (intraspecific variability data)/15,109 (human/chimpanzee ortholog data) proteins.

In general, we aimed to leverage all available public large-scale physical protein connectivity resources (current stable versions/builds as of mid-2019) which could be reliably and easily cross-linked with the protein evolutionary rate data, ending with the aforementioned three databases and five species, with the bulk of the results generated from the STRING human and mouse data. We were especially interested in the vertebrates because most of the previous research has been carried out in plants, yeast, and *C. elegans*.

To cross-link connectivity and variability data, three public resources were used: UniProt (UniProt: the universal protein knowledgebase 2017), USCS Genome Browser (Tyner et al. 2017), and Ensembl Genome Browser (Aken et al. 2016; Chen et al. 2010). After cross-referencing the proteins from the different databases, all proteins from all five species were assembled in the final dataset under uniform UniProt ID system. The final datasets can be found in Supplementary Material 2 (annotation) and Supplementary Materials 3, 4 (data). We used the total SNP number divided by the gene (mRNA) length as the “raw” evolutionary rate measure, and dN, dS, and dN/dS ratio as implemented in Aken et al. (2016), Chen et al. (2010), and Yang (1997). These were plotted (y-axis) against the protein connectivity (x-axis) in most of the results reported below. Python code for managing the datasets and visualizing the variability–connectivity relationships can be found in Supplementary Material 1.

### Computer Simulations

We used stochastic computer simulations of protein connectivity and protein variability to model three scenarios: no dependency between the former and the latter, linear dependency, and non-linear dependency. Subsequently, we investigated which of the three implemented scenarios fitted the real, observed, data the best.

To model protein connectivity, we used a protein network template: a connected network graph with nodes representing proteins, and edges—existing protein–protein interactions. During our simulation process, a new node is connected to the existing node *i* with the probability *p*_*i*_, where *k*_*i*_ is the number of connections of *i* (Albert and Barabási 2002):

$$ p_{i} = \frac{{k_{i} }}{{\sum\nolimits_{j} {k_{j} } }}. $$

In protein connectivity modeling function, we take the number of nodes as an input, and use this number to set up iterations. In each loop, we generate a new list of random values between 0 and 1. The length of the new list will increase by one in each loop, implying addition of a new node. The random number between 0 and 1 represents the probability of whether this new node is connected to the existing node. Next, we compare the random number with the probability assigned to the old node. If the former is less than the latter, these two nodes are connected by an edge, and the connectivity matrix is correspondingly updated. After the process is finished, the final matrix contains connectivity numbers (edge counts) for all nodes.

We tried the above algorithm with 1000–10,000 nodes. On a late model workstation, one 1000-node run takes ~ 2 s, whereas one 10,000-node run takes more than 30 min. We noticed that the protein connectivity distribution shape was essentially the same for any number of nodes, the only difference being in scaling along the protein connectivity axis. For example, to put it in biological context, for 1000 nodes, simulated protein connectivity scale was similar to that of the actually observed human Reactome “direct complex” subclass data. Because we were more interested in the distribution shape and patterns rather than the absolute values, we largely limited ourselves to 1000-node simulations throughout the study, which made the simulations computationally feasible without involving exotic computing resources. (However, results shown in Fig. 5 below were obtained with 10,000-node simulations).

For protein variability modeling, we first defined protein variability as the normalized total number of SNPs (i.e., divided by the mRNA length). We modeled the total SNP count dynamic over generations (assuming mutation neutrality) using binomial distribution (Sainudiin et al. 2007; Xu et al. 2012), setting the mRNA length at [100 × maximum SNP count].

In our first modeling scenario, we assumed that the protein connectivity network and protein variability are not coupled, evolving completely independently. Time, therefore, is the only variable linking protein connectivity and protein variability dynamics. Under this assumption, we take the number of nodes as an input, and set up the iterative process—in each loop (generation) we create a new list of random values following the binomial distribution. The list grows by one in each loop (addition of a new node). The binomial distribution-generated number is the number of SNPs in a new generation, for each node. Finally, we take inventory of all the SNPs across the generations, and return SNP vectors for all the nodes.

For the second and third modeling scenarios, we assume that protein connectivity and protein variability are not independent, and generate protein variability as a function of its connectivity. We will call it a variability–connectivity (V–C) function. (An obvious, and simplest, possible V–C function is the negative linear function, which is implemented in our second modeling scenario.) For the purposes of this study, we will limit ourselves to the negative (decreasing) functions. Under these conditions, we start by taking the number of nodes and the V–C function type as inputs in the iterative process. In each loop (generation), we use the method described in the preceding paragraph to generate a protein connectivity matrix. Using the updated matrix, and summing column-wise, we obtain a new protein connectivity vector after adding one new node. We then apply V–C function to this updated protein connectivity vector to estimate the probability of having one SNP count for the protein in this current generation. Then we use random sampling binomial distribution function to generate a vector of new SNPs for all the nodes in this generation; subsequently, we sum up SNPs counts for each generation, ascertain the maximum SNP count value, and multiply it by 100 to achieve uniform mRNA length. Finally, we divide the SNP counts by the mRNA length to calculate the protein variability. This procedure returns two vectors, one—containing protein connectivities, and another—protein variabilities. (It remains to note that we have tried at least 500 simulation runs for each of the three scenarios, and they proved to be exceedingly robust, with very little to practically no variation between the runs for our purposes.)

**Fig. 1:**
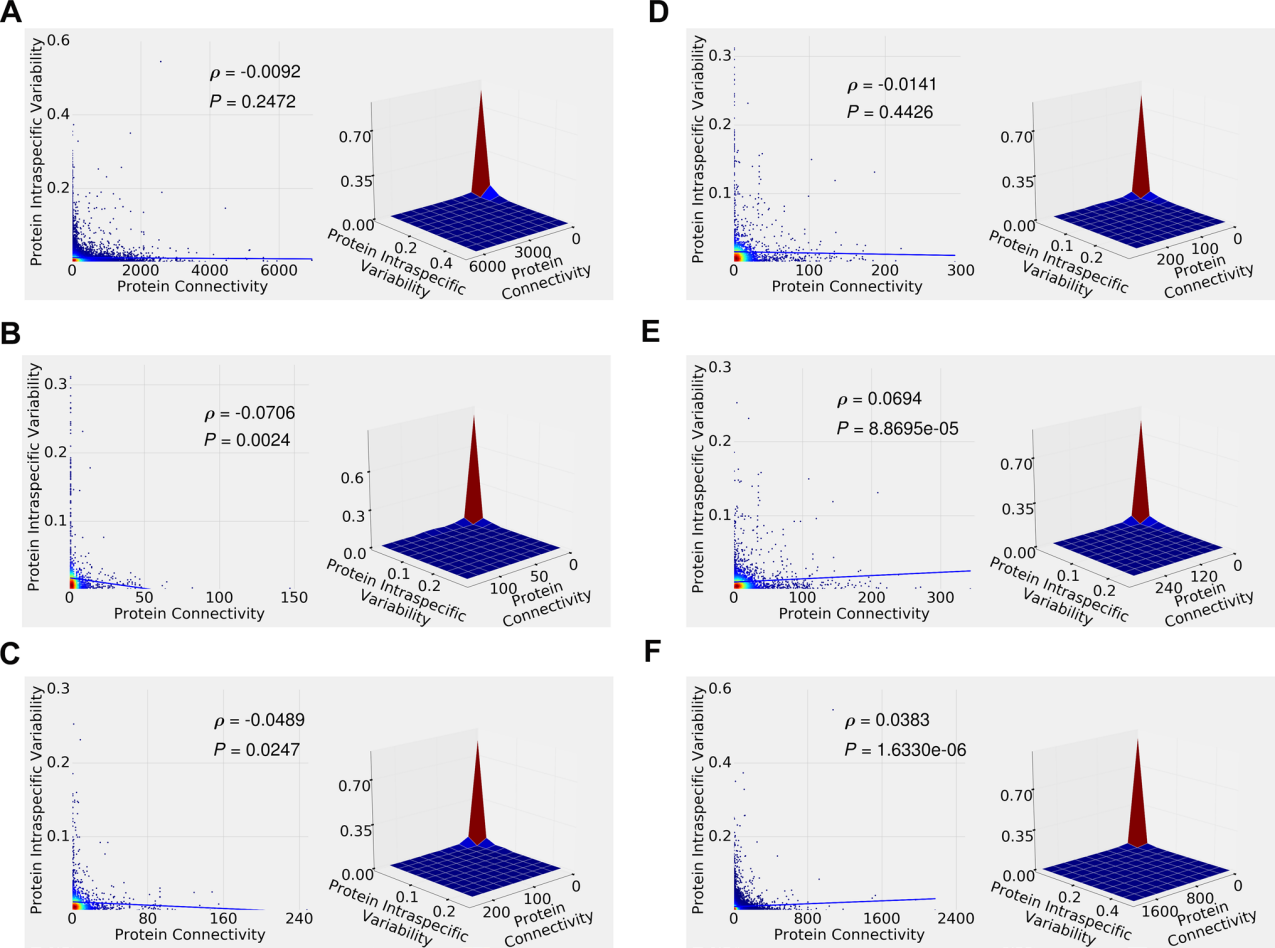
Density plots (left panes) and 3D surface plots (right panes) of human intraspecific protein variability versus protein connectivity. **a** STRING data. **b** Reactome “Direct Complex” data. **c** Reactome “Indirect Complex” data. **d** Reactome “Reaction” data. **e** Reactome “Neighboring Reaction” data. **f** APID data. Straight lines in the left panes depict fitted linear models. Spearman (non-parametric, rank) correlation coefficient/statistical significance (ρ and *P* value) are shown for each plot

The crucial question, and the cornerstone of the present study, is the choice of the V–C function(s). This study was partly motivated by a simple observation that the actual, observed, protein variability–connectivity patterns did not look particularly linear (regardless of whether the correlation was positive, negative, or non-existent). In fact, in our analyses (see Figs. 1, 3, Supplementary Figs. 2, 4, 6 in the "Results" section below) they looked rather “curvy.” Therefore, in addition to a linear V–C function, we have tried out a number of simple but non-linear functions. From the evolutionary standpoint, the exponential decay (or, negative exponential) function is a logical first choice. Indeed, it is well known (Sawyer and Hartl 1992) that the probability of fixation of an allele under selection decreases exponentially if negative selection is assumed. Here, we further hypothesize that the negative selection coefficient is linearly proportional to the protein’s connectivity, thus ending with the exponential decay V–C function as the simplest one fitting this evolutionary scenario.

Our goal was to show that a non-linear (but still simple) function might be a much better fit with the real data, and that this observation might help in resolving the variability–connectivity correlation (or absence thereof) conundrum. Of course, for each observed variability–connectivity dataset it is possible to over-parameterize the V–C functions to the extent that the fit will be asymptotically perfect (or at least much better than that obtained via a simple exponential decay V–C function), but such overfitting does not make much predictive modeling (or biological) sense. Eventually, with all these considerations in mind, we picked the negative linear V–C function for our second modeling scenario, and the exponential decay function for our third modeling scenario, with the probability of having a new SNP being, respectively, either

$$ a*c + b $$

or

$$ e^{ - k*c}, $$

where *c* is the protein connectivity. (We discuss the parameters (*a*, *b*, *k*) choice below in the "Results/Computer Simulation" section.) Python code for modeling all three scenarios can be found in Supplementary Material 1.

## Results

### Observed Data

**Fig. 2:**
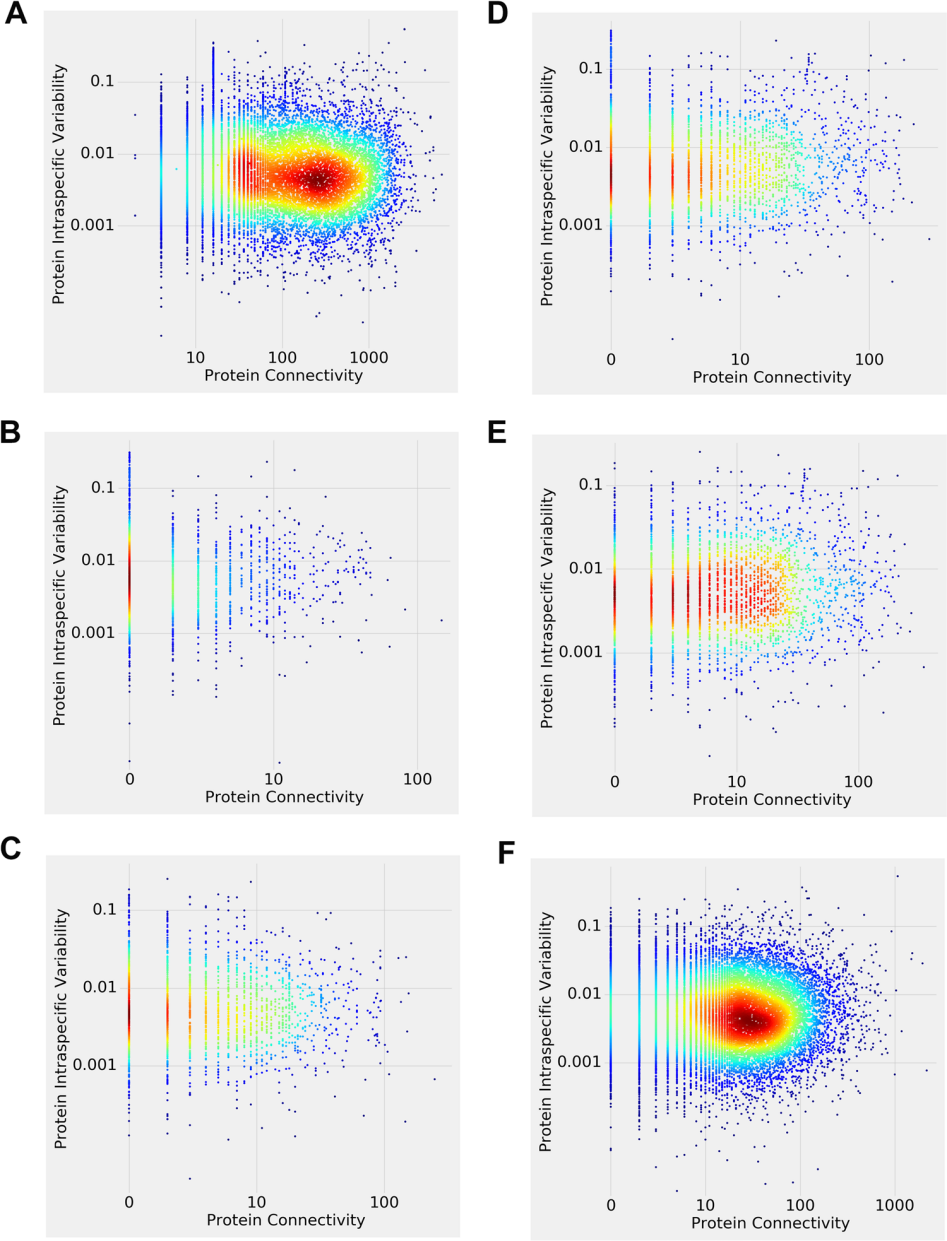
Density plots of human intraspecific protein variability versus protein connectivity, shown in the log–log scale to highlight the low variability–low connectivity areas. **a** STRING data. **b** Reactome “Direct Complex” data. **c** Reactome “Indirect Complex” data. **d** Reactome “Reaction” data. **e** Reactome “Neighboring Reaction” data. **f** APID data

Figure 1 depicts intraspecific protein variability versus protein connectivity relationships observed in the human STRING, Reactome, and APID data. Figure 1a, left pane, is a “heat map” visualization (density plot, with each point representing one gene) of the STRING data, with red color corresponding to the highest, and blue—lowest counts of the proteins with given connectivity and variability values. Figure 1a, right pane, is an alternative “3D surface plot” (joint probability distribution) visualization of the STRING data, where proteins were subdivided into ten equal-size bins (corresponding to 0.0–1.0 scale). It is clear from Fig. 1a that most human proteins have low connectivity and low variability (but there is a relative decrease in proteins with very low variability); very few have high connectivity and high variability; and some have high connectivity/low variability or low connectivity/high variability. Similar pattern is observed in the human Reactome data (Fig. 1b, “direct complex”; Fig. 1c, “indirect complex”; Fig. 1d, “reaction”, Fig. 1e, “neighboring reaction”) and human APID data (Fig. 1f). Figure 2 depicts the same data, but shown in the log–log scale, highlighting the low variability–low connectivity area. Here, a more nuanced relationship pattern emerges—notably, there is a relative paucity of the *very* low variability proteins. (Similarly, Supplementary Fig. 1 depicts the same data in the linear scale but “zoomed in,” to further highlight the low variability–low connectivity area.)

After fitting the straight lines for the linear model fit visualization, and computing the Spearman rank correlation coefficients, we observe insignificant negative correlation in the STRING (Fig. 1a) data, significant negative correlation in the Reactome “direct complex” (Fig. 1b) data, significant negative correlation in the Reactome “indirect complex” (Fig. 1c) data, insignificant negative correlation in the Reactome “reaction” (Fig. 1d) data, and significant positive correlation in the Reactome “neighboring reaction” (Fig. 1e) and APID (Fig. 1f) data. STRING and APID datasets have many more proteins than Reactome, and corresponding results are likely to be more robust. While the negative correlation observed in the STRING data (Fig. 1a) is weak and insignificant, and the positive correlation observed in the APID data (Fig. 1f) is significant, the actual distributions, when not reduced to the simple linear models (or rank correlation statistics), are quite similar between the two (Fig. 1a, f, 2a, f; Supplemental Fig. 1a, f). This suggests that “compressing” the full distributional information into the single-number linear (or rank) correlation coefficients might not be the best tactic for comparing and/or contrasting the protein variability–connectivity distributions. (Instead, distributional distance-based approaches are explored below in the "Results/Computer Simulation" section.)

Supplementary Fig. 2 (and Supplementary Fig. 3, same data but in the log–log scale) depicts dN/dS ratio versus protein connectivity relationship observed in human/chimpanzee orthologs (STRING, Reactome and APID databases). Here, we see broadly the same distribution shapes as in Fig. 1. However, we observe significant negative variability–connectivity correlation in the larger STRING and APID datasets (and no strongly discernible trend across the smaller Reactome datasets). We conclude that proteins with high connectivity tend to show lower dN/dS values [reflecting stronger purifying (negative) selection]. Supplementary Figs. 4 and 6 (and Supplementary Figs. 5 and 7, same data but in the log–log scale) show dN versus protein connectivity and dS versus protein connectivity relationships, respectively. The same distributional shapes and patterns remain. So does the tendency towards negative variability–connectivity correlation, more pronounced with the dN data.

**Fig. 3:**
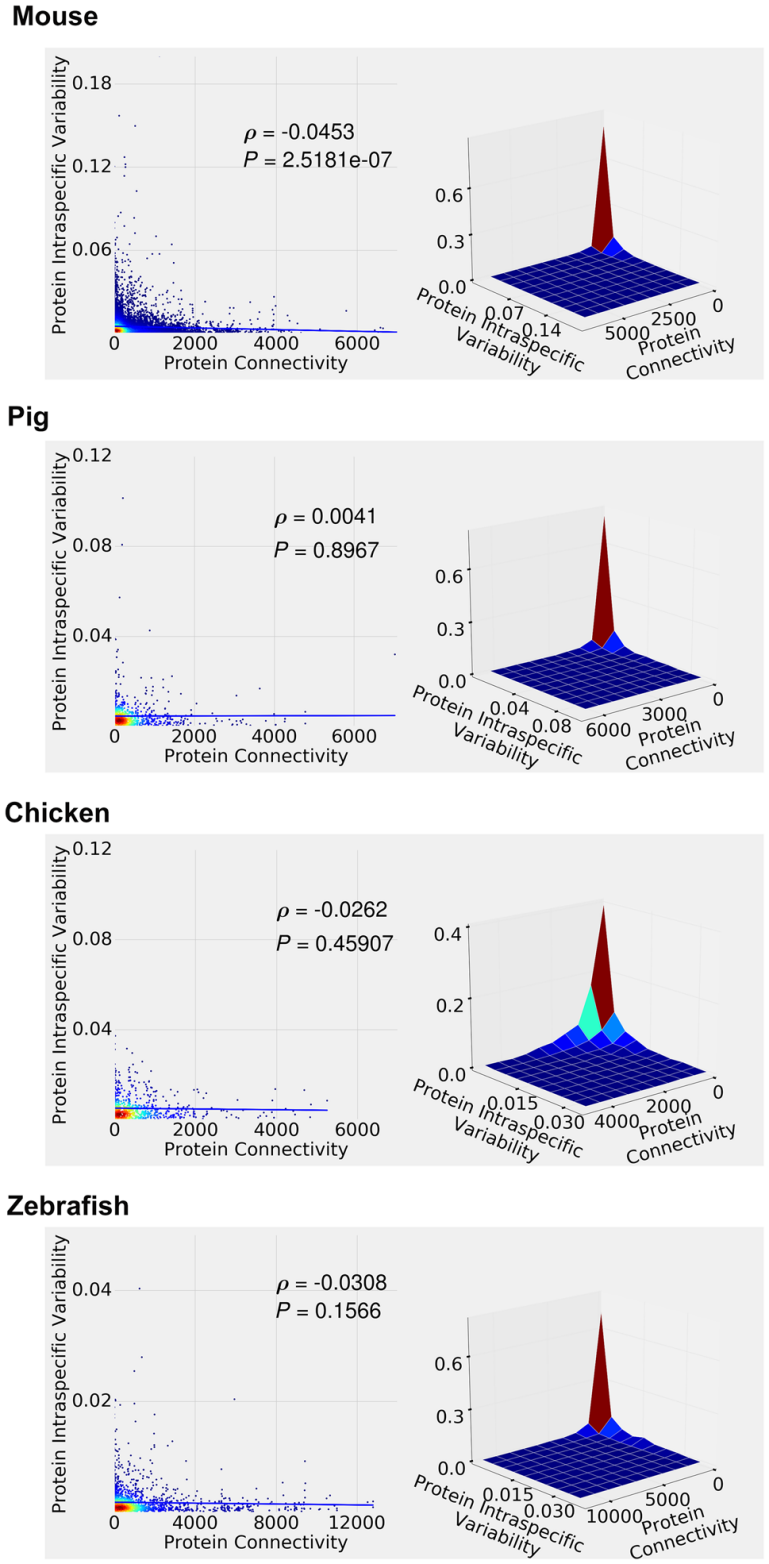
Density plots (left panes) and 3D surface plots (right panes) of mouse, pig, chicken, and zebrafish intraspecific protein variability versus protein connectivity (STRING data)

Figure 3 (and Supplementary Fig. 8, same data but in the log–log scale) depicts intraspecific protein variability versus protein connectivity relationships observed in mouse, pig, chicken, and zebrafish (STRING database). While the shapes and relative densities vary, overall the five distributions are similar to each other and to the human distribution (see Fig. 1a). The differences between the distributions might have less to do with the interspecific differences than with the way the datasets are assembled and curated. Only the mouse data show significant negative correlation; other species do not reveal significant correlation coefficients.

Supplementary Fig. 9 (and Supplementary Fig. 10, same data but in the log–log scale) depicts dN/dS, dN, and dS versus protein connectivity relationships observed in mouse/rat orthologs (STRING database). (We have limited our interspecific analyses to human/chimpanzee and mouse/rat, as it was difficult to obtain sizable confirmed ortholog datasets for the remaining species.) The dN/dS and dN patterns remain similar to the ones observed in human/chimpanzee orthologs (Supplementary Figs. 2, 4). The dS pattern, however, is somewhat different. There are very few proteins with low dS, reflecting comparatively high divergence between mouse and rat. It is reasonable to assume that the patterns of dS–connectivity relationships would, in general, become less pronounced with increasingly more divergent sequences, especially if the synonymous sites are not under significant selection pressure. Just as with the human/chimpanzee ortholog data (Supplementary Figs. 2, 4, 6), we observe the general trend towards negative correlation (here significant for all three datasets, dN/dS, dN, and dS), which is more pronounced compared to the mouse intraspecific variability data (Fig. 3a).

All of the above results (shown in Figs. 1, 3; Supplemental Figs. 2, 4, 6, 9) are summarized in Table 1 below. We conclude that, in general, there is an overall tendency towards the negative protein variability–connectivity correlation in the orthologs (interspecific variability) data. It is more pronounced for the dN/dS and dN than for the dS data, arguably reflecting selection pressure. The intraspecific variability results are ambiguous, ranging from the positive correlation in some larger human databases (APID) to virtually no correlation in other larger human databases (STRING) to the negative correlation in mouse (STRING database) to no significant correlation in pig, chicken, and zebrafish (STRING database). The distributional shapes, however, suggest a typical and distinct variability–connectivity relationship pattern, largely invariant between the different species, databases, and analyses.

**Table 1:** Spearman correlation coefficient and statistical significance (ρ and *P* value) for protein variability–connectivity relationships shown in Figs. 1, 3 and Supplemental Figs. 2, 4, 6, 9

	ρ	*P* value
Human intraspecific protein variability versus protein connectivity (Fig. 1)		
STRING	− 0.0092	0.2472
Reactome direct complex	− 0.0706	0.0024
Reactome indirect complex	− 0.0489	0.0247
Reactome reaction	− 0.0141	0.4426
Reactome neighboring reaction	0.0694	8.8695e−05
APID	0.0383	1.6330e−06
Human/chimpanzee ortholog protein dN/dS ratio versus protein connectivity (Supplemental Fig. 2)		
STRING	− 0.1058	7.5334e−39
Reactome direct complex	− 0.0226	0.3475
Reactome indirect complex	− 0.0527	0.0184
Reactome reaction	− 0.0139	0.4592
Reactome neighboring reaction	0.0076	0.6744
APID	− 0.0750	2.5481e−20
Human/chimpanzee ortholog protein dN versus protein connectivity (Supplemental Fig. 4)		
STRING	− 0.0639	3.5349e−15
Reactome direct complex	− 0.0279	0.2464
Reactome indirect complex	− 0.0403	0.0714
Reactome reaction	− 0.0226	0.2298
Reactome neighboring reaction	0.0110	0.5407
APID	− 0.0388	1.8759e−06
Human/chimpanzee ortholog protein dS versus protein connectivity (Supplemental Fig. 6)		
STRING	− 0.0168	0.0393
Reactome direct complex	− 0.0338	0.1606
Reactome indirect complex	− 0.0214	0.3386
Reactome reaction	− 0.0167	0.3765
Reactome neighboring reaction	0.0007	0.9677
APID	− 0.0127	0.1194
Mouse, pig, chicken, and zebrafish intraspecific protein variability versus protein connectivity (Fig. 3)		
Mouse	− 0.0453	2.5181e−07
Pig	0.0041	0.8967
Chicken	− 0.0262	0.45907
Zebrafish	− 0.0308	0.1566
Mouse/rat ortholog protein variability versus protein connectivity (Supplemental Fig. 9)		
dN/dS ratio	− 0.1638	3.7644e−92
dN	− 0.1590	7.3377e−87
dS	− 0.1036	1.2849e−37

### Computer Simulation Results

**Fig. 4:**
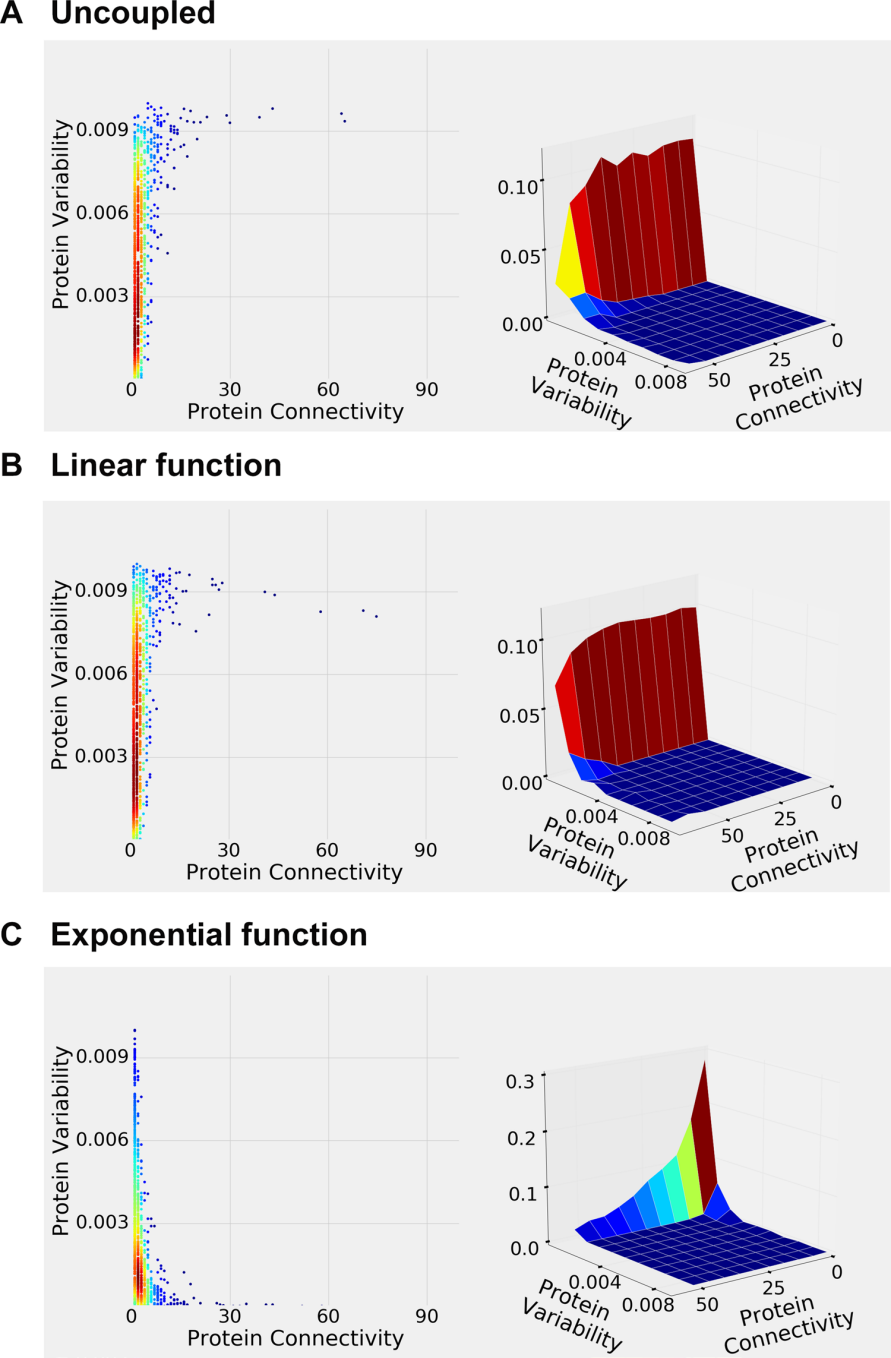
Density plots (left panes) and 3D surface plots (right panes) of simulated protein variability versus protein connectivity. **a** protein variability and connectivity are modeled independently (see “first modeling scenario” in Methods). **b** protein variability and connectivity are linked via negative linear function (second modeling scenario). **c** protein variability and connectivity are linked via exponential decay function (third modeling scenario)

Figure 4a illustrates our first modeling scenario (see Methods), in which protein connectivity and variability were modeled independently. Low connectivity proteins display just about any variability; high connectivity proteins tend to display high variability. In general, this plot is very dissimilar to the distribution shapes in Figs. 1, 3 and Supplementary Figs. 2, 4, 6, 9 (we will explicitly quantify the extent of the dissimilarities in Table 2 below, after introducing two distributional distance measures).

We now proceed with two V–C functions that “couple” variability and connectivity throughout the stochastic simulation modeling process. Figure 4b illustrates our second modeling scenario (negative linear V–C function) and Fig. 4c third modeling scenario (exponential decay V–C function). The actual V–C functions in Fig. 4 are

$$ a*c + b, $$

where *a* = − 0.004, *b* = 1, and *c* is the protein connectivity (Fig. 4b), and

$$ e^{ - k*c}, $$

where *k *= 1, and *c* is the protein connectivity (Fig. 4c). (Supplementary Fig. 11A–C depicts the same data as in Fig. 4a–c, but in the log–log scale.) We have tried various V–C function parameter values (*a*, *b*, *k*) while aiming to keep the resulting counts and values in the realistic range. Corresponding simulations are shown in the Supplementary Material 5 (negative linear) and Supplementary Material 6 (exponential decay). The former (negative linear) results in the largely alike distributions, of which the distribution shown in Fig. 4b is representative. Again, it is dissimilar to the distribution shapes observed in the real data. The latter (exponential decay) range from the convergence to the negative linear (when *k *~ = 0) to the “compression” into very few unique data points (when *k* > 4)—the distribution shown in Fig. 4c (*k* = 1) is representative of the distributions with mid-*k* values, and at a first glance is not dissimilar to the ones observed in the real data (Figs. 1, 3; Supplementary Figs. 2, 4, 6, 9).

**Fig. 5:**
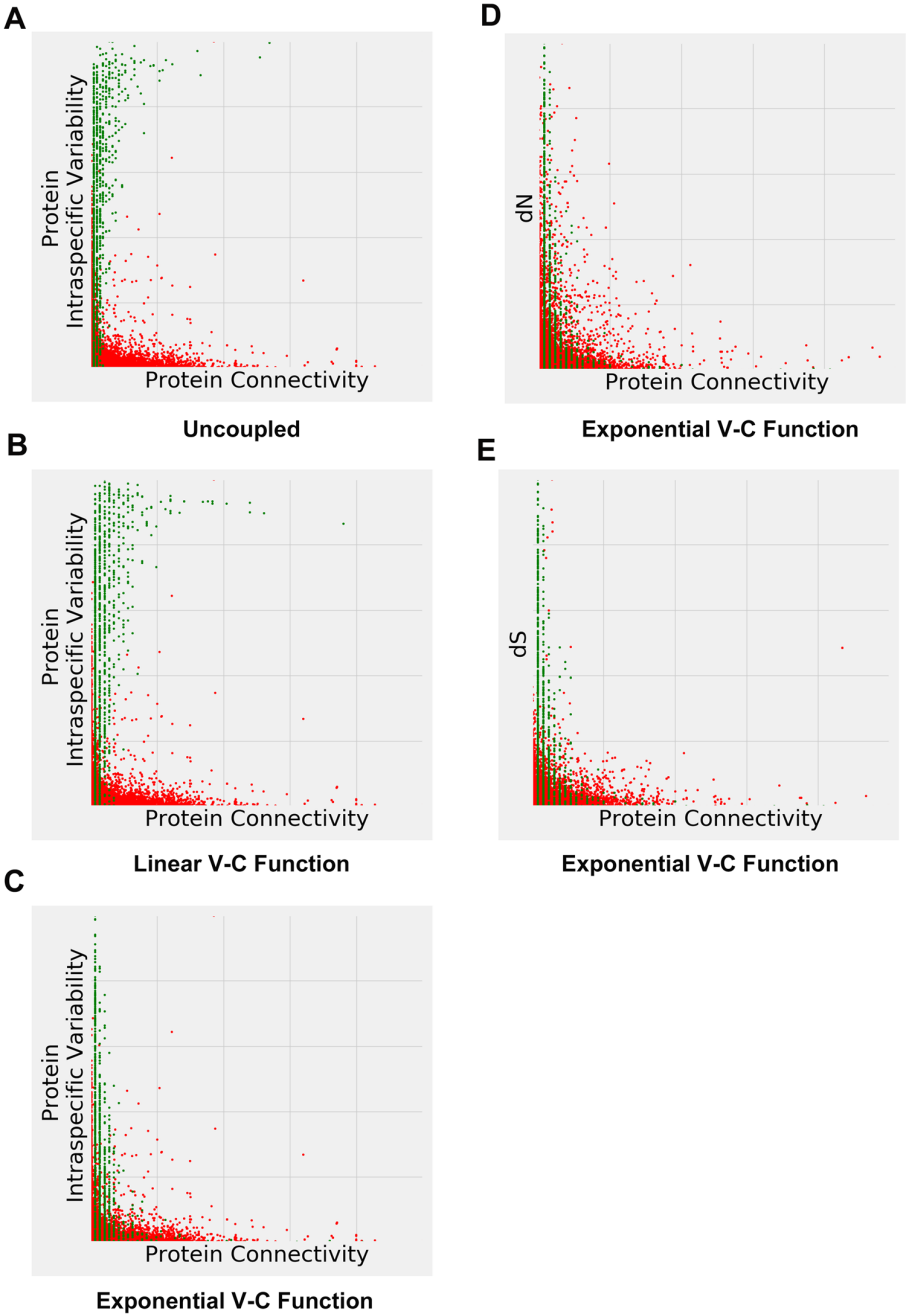
Scatter plots of observed human data from STRING database (red dots) superimposed on the simulation results (green dots). **a** Intraspecific variability data (red dots); protein variability and connectivity are modeled independently (green dots). **b** Intraspecific variability data (red dots); protein variability and protein connectivity are linked via negative linear function (green dots). **c** Intraspecific variability data (red dots); protein variability and connectivity are linked via exponential decay function (green dots). **d** Human/chimpanzee ortholog protein dN values (red dots); protein variability and connectivity are linked via exponential decay function (green dots). **e** Human/chimpanzee ortholog protein dS values (red dots); protein variability and connectivity are linked via exponential decay function (green dots) (Color figure online)

We will now evaluate the actual similarity/dissimilarity between the real data and the distributions generated by the uncoupled, negative linear, and exponential decay V–C functions. It is difficult to do so by directly observing the plots, largely because of the different protein connectivity scaling (depending on the species, database size, type of protein–protein interaction, and whether it is the observed data, or a 10,000-node simulation experiment). Therefore, to illustrate on the human STRING data example, in Fig. 5 we re-scale protein connectivity to the single common [0 (min connectivity value)—1 (max connectivity value)] range, and scatter-plot both observed STRING human data [red dots, same data points as in Fig. 1a (intraspecific variability), Supplemental Figs. 4a (dN) and 6a (dS)] and simulated data (green dots, same data points as in Fig. 4). Figure 5a depicts human intraspecific variability/no V–C coupling combination; Fig. 5b depicts human intraspecific variability/linear V–C function combination; Fig. 5c—human intraspecific variability/exponential decay V–C function combination; Fig. 5d—human–chimpanzee ortholog protein dN/exponential decay V–C function combination; Fig. 5e—human-chimpanzee ortholog protein dS/exponential decay V–C function combination. (Supplementary Fig. 12a–e depicts the same data as in Fig. 5a–e, but in the log–log scale.)

The goal of this study was not necessarily to find the V–C function that would lead to the perfect fit to the observed data, but rather to show that (i) no V–C function at all might not lead to a good fit (e.g., Fig. 5a), (ii) negative linear V–C function might not lead to a good fit (e.g., Fig. 5b), (iii) some other simple V–C function (possibly a “curvy”, convex, decreasing one) might lead to a better fit. It appears that the exponential decay V–C function works reasonably well, leading to a better fit with the real data (e.g., Fig. 5c–e). This is consistent with the dynamics of the probability of allele fixation under the negative selection (Sawyer and Hartl 1992).

Finally, we will use distributional distance measures (Energy Distance, ED, and Earth Mover’s Distance, EMD) to quantify the distributional similarities/dissimilarities between the real and the simulated data. Both ED and EMD generalize easily to the multidimensional data and are more appropriate than the “standard” Kolmogorov–Smirnov distance for the distributions that are known to be possibly significantly dissimilar and/or have relatively heavy tails/numerous outliers; both distances are scale sensitive, range from zero to infinity, and are proportional to the empirical distribution sample sizes (Rizzo and Szekely 2016; Rubner et al. 1998). Table 2 summarizes the ED and EMD values for the human STRING data comparisons (as plotted in Fig. 5). Both metrics suggest that the exponential decay V–C function leads to a better fit (lower distributional distances) with the real data than the negative linear V–C function or no V–C function. (To provide the sense of scale, EMD is roughly equal to the area between the two distributions’ empirical CDFs, or cumulative distribution functions—therefore, a difference between, for example, EMD of 360.83 and 116.1, as shown in Table 2, is highly significant; similarly, ED is a linear function of the Cramer Distance, with the same scale sensitivity). This is consistent throughout the realistic range of the negative linear and exponential decay functions’ parameter values (Supplementary Materials 5, 6). To further demonstrate that even the “best” possible negative linear model fit is still inferior to the exponential decay model fit, we have obtained the ED values (between the real human STRING data and the simulated data) for the widest possible range (that fits into our simulation framework) of the negative linear V–C function parameters (*a* and *b*), averaged over 100 simulations for each parameter combination (Supplementary Material 7; we did not obtain the EMD values due to the EMD being computationally much more demanding). Even the lowest ED value (420.3298 in Supplementary Material 7) was significantly higher than the typical (*k *=1, non-optimized) ED value for the exponential decay model data (231.70 in Table 2). (Python code for implementing ED, EMD, and the above simulations can be found in Supplementary Material 1.)

**Table 2:** Energy Distance (ED) and Earth Mover’s Distance (EMD) between the observed (human STRING data) and simulated distributions (*a *=− 0.004, *b *=1 for the negative linear V–C function, *k *=1 for the exponential decay V–C function), averaged over 100 simulation replications for ED and 5 simulation replications for EMD

	Exponential decay V–C function	Negative linear V–C function	V–C uncoupled
ED			
STRING Intraspecific variability	231.70 (Fig. 5c)	584.07 (Fig. 5b)	576.85 (Fig. 5a)
STRING dN	221.77 (Fig. 5d)	570.20	536.28
STRING dS	227.82 (Fig. 5e)	597.37	562.61
EMD			
STRING Intraspecific variability	116.11 (Fig. 5c)	360.83 (Fig. 5b)	336.99 (Fig. 5a)
STRING dN	126.43 (Fig. 5d)	507.96	473.25
STRING dS	132.03 (Fig. 5e)	373.90	351.21

## Discussion

The main conclusions of this study are threefold: First, when analyzed in a “traditional” fashion (linear models and/or rank correlation), large-scale genomic vertebrate datasets suggest that there is a tendency towards weak but often significant negative correlation between the protein connectivity and interspecific variability (Supplementary Figs. 2, 4, 6, 9, Table 1). The intraspecific variability results (Figs. 1, 3; Table 1), on the other hand, are ambiguous. Second, the patterns of protein variability–connectivity relationships, while strongly pronounced, are not linear (they tend to have a convex decreasing shape, combined with the relative paucity of the very low variability proteins), and probably should not be evaluated by fitting linear models. The latter tends to, at best, underestimate the variability–connectivity association effect. At worst, they might lead to the “false negatives,” suggesting non-existent or marginal correlation where there is in fact a strong non-random pattern. Third, simulation experiments can be used to generate patterns similar to the ones observed in the real data. Coupling variability and connectivity during the simulation modeling process via, for example, an exponential decay function produces a better distributional fit (between the simulated and observed variability–connectivity distributions) compared to the negative linear function or no coupling at all.

Of course, while our modeling results approximate the real data patterns sufficiently well (Fig. 5c–e), there are still notable discrepancies. More broadly, protein variability is affected by many factors other than PPIs—expression levels and patterns, protein age, protein length and structure, gene structure, chromatin factors, epigenetic factors in general, etc. In future, we plan to expand our simulation framework by gradually introducing more (and more biologically motivated) parameters. One possible research direction is to “fix” the PPI network (along the lines of the real data) and simulate the protein variability over the fixed network topology and parameters. This would be especially fitting for the interspecific (divergence) data, as the simulation and “real” evolution timescales would be better aligned—there is significant evidence that PPIs, on average, evolve more slowly than protein sequences (Ghadie et al. 2017).

Another interesting aspect is classifying PPIs, and/or corresponding proteins, into distinct subgroups. Recent literature (Biswas et al. 2017; Pang et al. 2016) suggests that, in general, different subgroups might show very different selection/variability patterns. For example, there is evolutionary rate heterogeneity between the proteins associated with human PPI single-interface “hubs” and multi-interface “hubs” (Biswas et al. 2017). Similarly, proteins associated with human PPI “hubs/non-hubs,” “bottlenecks/non-bottlenecks,” and various combinations thereof show different evolutionary rates and patterns (Pang et al. 2016). We feel that an effort to establish some “formal,” universally accepted, standard for further subgroup classification is long overdue, and so are the subgroup-specific protein variability analyses (as opposed to pooling all the proteins together, on a continuous spectrum, as was done in this and majority of the preceding studies).

On a genomic level, high connectivity has been shown to be correlated with conserved synteny (preserved gene order across different species); while the association was found to be moderate, connectivity still possessed predictive value (independently of orthology) with respect to conserved synteny (Kirk et al. 2017b). The next level is large-scale genomic aberrations (to which chromosome-specific PPI patterns can be linked) (Kirk et al. 2017a). This and other genome-wide effects, such as PPIs being mediated by multiple sequence regions (Ghadie et al. 2017), add a whole new (higher) plane of biological structure/regulation to the picture, and simulating PPI evolution on a genomic level is undoubtedly a yet another intriguing research direction.

The results of our study dovetail, in a complementary way, with the recent work by Alvarez-Ponce et al. (2017), the goal of which was to rank and compare various components (including PPI characteristics and expression parameters) contributing to protein evolutionary rate variation, using human (human/mouse orthologs) and other species (*D. melanogaster/D. yakuba, C. elegans/C. briggsae,* and *S. cerevisiae/S. paradoxus* orthologs) data. Correlation (or partial correlation) was used to assess the “strength” of all components; they were subsequently integrated (and compared) in the principal component analysis framework. Eventually, PPI/centrality-related components were found to be at least as important as the gene expression-related ones in contributing (independently) to dN and dN/dS. The effect was most pronounced in *H. sapiens*. Interestingly, while the protein interaction/centrality results in Alvarez-Ponce et al. (2017) (Figs. 1a, 3a, b in Alvarez-Ponce et al. 2017) are very similar to ours, using correlation to measure the protein interaction/centrality component’s strength probably *downplays* the importance of the protein interaction/centrality component. An approach similar to the one employed in our study (using distributional proximity metrics instead of correlation coefficients) would have arguably strengthened Alvarez-Ponce, Feyertag, and Chakraborty’s main conclusion (namely, that network centrality has substantial independent impact on the rates of protein evolution). We should note here that our analyses did not include expression levels and patterns (or any components other than protein connectivity)—therefore, at this time, we would rather not speculate on the correlation (or independence) of PPI-related factors and expression-related factors contributing to the rates of protein evolution—leaving it to the future, multi-component, analyses and simulations.

In closing, we would like to posit the question: why are we so curious about the interplay of protein connectivity and variability to begin with? In addition to the obvious aspects, discussed at some length throughout this manuscript and elsewhere, there is also an issue of a purely pragmatic significance:

Picture a typical large-scale biomedical research study in which large sets of candidate genes (proteins), to be considered for the individual follow-up studies, are generated. How do we prioritize/rank them for the future research? In other words, can we come up with the multidimensional measure of how “interesting,” or “important,” a specific candidate gene (protein) is? PPI data (in particular, connectivity or centrality) should probably factor into it. So should expression patterns, selection pressures, etc. Recently, Zhang, Xiao, and Hu developed such an integrated metric (“orthogonal centrality measure”) (Zhang et al. 2018) to predict “essential” (on organismal level) proteins. While predicting interesting, or important, proteins is of course different (for one, we do not have the known class labels for interesting/important proteins, as we do for essential ones), a conceptually similar integrative approach is probably the most viable way to combine both PPI and evolutionary factors in a single predictive analytic framework. Such framework should also include expression levels and patterns and other components (Alvarez-Ponce et al. 2017). As we have illustrated throughout this study, the relationship between PPI and variability is a complicated and nuanced one. Incorporating both into a single protein “importance” metric is a worthwhile goal and a promising research direction to be pursued further.

## Electronic supplementary material

Below is the link to the electronic supplementary material.

Supplementary material 1 (TIFF 5320 kb). Supplementary Fig. 1. Density plots of human intraspecific protein variability *vs.* protein connectivity, “zoomed in” to highlight the low variability – low connectivity areas. (A) STRING data. (B) Reactome “Direct Complex” data. (C) Reactome “Indirect Complex” data. (D) Reactome “Reaction” data. (E) Reactome “Neighboring Reaction” data. (F) APID data

Supplementary material 2 (TIFF 6218 kb). Supplementary Fig. 2 Density plots (left panes) and 3D surface plots (right panes) of human / chimpanzee ortholog protein dN/dS ratio *vs.* protein connectivity. (A) STRING data. (B) Reactome “Direct Complex” data. (C) Reactome “Indirect Complex” data. (D) Reactome “Reaction” data. (E) Reactome “Neighboring Reaction” data. (F) APID data

Supplementary material 3 (TIFF 4327 kb). Supplementary Fig. 3 Density plots of human / chimpanzee ortholog protein dN/dS ratio *vs.* protein connectivity, shown in the log-log scale. (A) STRING data. (B) Reactome “Direct Complex” data. (C) Reactome “Indirect Complex” data. (D) Reactome “Reaction” data. (E) Reactome “Neighboring Reaction” data. (F) APID data

Supplementary material 4 (TIFF 6293 kb). Supplementary Fig. 4 Density plots (left panes) and 3D surface plots (right panes) of human / chimpanzee ortholog protein dN values *vs.* protein connectivity. (A) STRING data. (B) Reactome “Direct Complex” data. (C) Reactome “Indirect Complex” data. (D) Reactome “Reaction” data. (E) Reactome “Neighboring Reaction” data. (F) APID data

Supplementary material 5 (TIFF 4476 kb). Supplementary Fig. 5 Density plots of human / chimpanzee ortholog protein dN values *vs.* protein connectivity, shown in the log-log scale. (A) STRING data. (B) Reactome “Direct Complex” data. (C) Reactome “Indirect Complex” data. (D) Reactome “Reaction” data. (E) Reactome “Neighboring Reaction” data. (F) APID data

Supplementary material 6 (TIFF 6120 kb). Supplementary Fig. 6 Density plots (left panes) and 3D surface plots (right panes) of human / chimpanzee ortholog protein dS values *vs.* protein connectivity. (A) STRING data. (B) Reactome “Direct Complex” data. (C) Reactome “Indirect Complex” data. (D) Reactome “Reaction” data. (E) Reactome “Neighboring Reaction” data. (F) APID data

Supplementary material 7 (TIFF 4187 kb). Supplementary Fig. 7 Density plots of human / chimpanzee ortholog protein dS values *vs.* protein connectivity, shown in the log-log scale. (A) STRING data. (B) Reactome “Direct Complex” data. (C) Reactome “Indirect Complex” data. (D) Reactome “Reaction” data. (E) Reactome “Neighboring Reaction” data. (F) APID data

Supplementary material 8 (TIFF 3464 kb). Supplementary Fig. 8 Density plots of mouse, pig, chicken and zebrafish intraspecific protein variability *vs.* protein connectivity (STRING data), shown in the log-log scale

Supplementary material 9 (TIFF 3762 kb). Supplementary Fig. 9 Density plots (left panes) and 3D surface plots (right panes) of mouse / rat ortholog protein variability *vs.* protein connectivity (STRING data). (A) dN/dS ratio *vs.* protein connectivity. (B) dN *vs.* protein connectivity. (C) dS *vs.* protein connectivity

Supplementary material 10 (TIFF 3498 kb). Supplementary Fig. 10 Density plots of mouse / rat ortholog protein variability *vs.* protein connectivity (STRING data), shown in the log-log scale. (A) dN/dS ratio *vs.* protein connectivity. (B) dN *vs.* protein connectivity. (C) dS *vs.* protein connectivity

Supplementary material 11 (TIFF 492 kb). Supplementary Fig. 11 Density plots of simulated protein variability *vs.* protein connectivity, shown in the log-log scale. (A) protein variability and connectivity are modeled independently (see “first modeling scenario” in Methods). (B) protein variability and connectivity are linked *via* negative linear function (second modeling scenario). (C) protein variability and connectivity are linked *via* exponential decay function (third modeling scenario)

Supplementary material 12 (TIFF 2052 kb). Supplementary Fig. 12 Scatter plots of observed human data from STRING database (red dots) superimposed on the simulation results (green dots), shown in the log-log scale. (A) intraspecific variability data (red dots); protein variability and connectivity are modeled independently (green dots). (B) intraspecific variability data (red dots); protein variability and protein connectivity are linked *via* negative linear function (green dots). (C) intraspecific variability data (red dots); protein variability and connectivity are linked *via* exponential decay function (green dots). (D) human / chimpanzee ortholog protein dN values (red dots); protein variability and connectivity are linked *via* exponential decay function (green dots). (E) human / chimpanzee ortholog protein dS values (red dots); protein variability and connectivity are linked *via* exponential decay function (green dots)

Supplementary material 13 (PDF 100 kb). Supplementary Material 1. Python code for the analyses / simulations in this study

Supplementary material 14 (PDF 36 kb). Supplementary Material 2. Annotation for the data files

Supplementary material 15 (RAR 7284 kb). Supplementary Material 3. Datasets part 1 (RAR, compressed ASCII)

Supplementary material 16 (RAR 7856 kb). Supplementary Material 4. Datasets part 2 (RAR, compressed ASCII)

Supplementary material 17 (PDF 1795 kb). Supplementary Material 5. Simulation results (negative linear V-C function). Density plots (left panes) and 3D surface plots (right panes) of simulated protein variability *vs.* protein connectivity, with protein variability and connectivity linked *via* negative linear function (second modeling scenario). Intercept and slope parameter values are shown next to each simulation plot

Supplementary material 18 (PDF 1341 kb). Supplementary Material 6. Simulation results (exponential decay V-C function). Density plots (left panes) and 3D surface plots (right panes) of simulated protein variability *vs.* protein connectivity, with protein variability and connectivity linked *via* exponential decay function (third modeling scenario). Exponential decay constant parameter value is shown next to each simulation plot

Supplementary material 19 (PDF 86 kb). Supplementary Material 7. ED measure (distributional distance) between the simulated protein variability *vs.* protein connectivity distribution (with protein variability and connectivity linked *via* negative linear function, second modeling scenario) and observed human data from STRING database, shown across a wide range of negative linear function parameters (*a* and *b*), averaged over 100 simulations for each parameter combination. Simulations were carried out for 15903 nodes, to match exactly with the human STRING data. Darker orange color indicates higher ED values (more distributional divergence), less saturated yellow color --- lower ED values
