# Supplementary material for: Dependency Between Protein–Protein Interactions and Protein Variability and Evolutionary Rates in Vertebrates: Observed Relationships and Stochastic Modeling
Source: J Mol Evol. 2019 Jul 13;87(4):184–98. doi: 10.1007/s00239-019-09899-z (PMC6658588; doi:10.1007/s00239-019-09899-z)
Supplement: Supplementary file 13 — Supplementary material 13 (PDF 100 kb). Supplementary Material 1. Python code for the analyses / simulations in this study [file 239_2019_9899_MOESM13_ESM.pdf]

```
''' variability_connectivity_plot.py'''

import numpy as np
import matplotlib.pyplot as plt

'''
snp_file = 'ensemble_snp_4_13_first_try.txt'
connect_file = 'STRING_binding_connectivity_8_25_18.txt'
'''

def get_snp_connect_list(connect_file, snp_file):
    readfile_dnds = open(snp_file)
    name1 = []
    dnds_list = []
    for line in readfile_dnds:
        line = line. strip()
        split_list = line.split()
        protein = split_list[0]
        dnds = float(split_list[1])
        name1.append(protein)
        dnds_list.append(dnds)
    print('SNP data contains ', len(name1), ' proteins')
    name2 = []
    connect_list = []
    con_file = open(connect_file)
    for item in con_file:
        item = item.strip()
        split_item = item.split()
        protein_name = split_item[0]
        connect = int(split_item[1])
        name2.append(protein_name)
        connect_list.append(connect)
    print('connectivity data contains ', len(name2), ' proteins')
    common = list(set(name1) & set(name2))
    #print(common)
    index_list1 = find_index(name1, common)

    y = np.take(dnds_list, index_list1)
    index_list2 = find_index(name2, common)
    x = np.take(connect_list, index_list2)
    print('total found ', len(x))
    print('connectivity: lenth ',len(x), 'max ', max(x), 'min ', min(x),
'mean ', np.mean(x), 'me
dian ', np.median(x))
    print('varibility: lenth ',len(y), 'max ', max(y), 'min ', min(y),
'mean ', np.mean(y), 'medi
an ', np.median(y))

    return x,y

def get_dnds_connect_list(connect_file, dnds_file):
```

```
    readfile_dnds = open(dnds_file)
    name1 = []
    dnds_list = []
    for line in readfile_dnds:
        line = line. strip()
        split_list = line.split()
        protein = split_list[0]
        dnds = float(split_list[1])
        name1.append(protein)
        dnds_list.append(dnds)
    print('ensembl dnds data contains ', len(name1), ' proteins')
    name2 = []
    connect_list = []
    con_file = open(connect_file)
    for item in con_file:
        item = item.strip()
        split_item = item.split()
        protein_name = split_item[0]
        connect = int(split_item[1])
        name2.append(protein_name)
        connect_list.append(connect)
    print('connectivity data contains ', len(name2), ' proteins')
    common = list(set(name1) & set(name2))
    #print(common)
    index_list1 = find_index(name1, common)

    y = np.take(dnds_list, index_list1)
    index_list2 = find_index(name2, common)
    x = np.take(connect_list, index_list2)
    print('total found ', len(x))
    return x,y

'''   one_stop_c_v.py    '''


import numpy as np
from collections import Counter
import matplotlib.pyplot as plt
from scipy.stats import gaussian_kde
import modeling_join_probability as mjp
from mpl_toolkits.mplot3d import Axes3D
import sys
from matplotlib import cm

def readfile_to_list(filename):
    readfile = open (filename)
    ProteinList = []
    for line in readfile:
        line = line.strip()
        split_list = line.split()
        protein_name = split_list[0]
        value = split_list[1]
        temp = []
        temp.append(protein_name)
```

```
        temp.append(value)
        ProteinList.append(temp)
    return ProteinList

#------------------------- get SNP count -------------------------------
-------

#read snp data from downloaded Ensembl database
import numpy as np

def make_address(index_no):
    address =
'/home/xicwang/DnDs_project_1/ensembl_SNP_4_12_2019/ensembl_snp_data_down
load_c1_c2
2/mart_export_C'
    address += str(index_no)
    address += '.txt'
    return address

def read_to_list(filename):
    a = open(filename)
    geneName_dic = {}
    count = 0
    for line in a:
        count +=1
        if count >=2:
            line = line. strip()
            split_list = line.split()
            #print(split_list)
            #if len(split_list) == 5:
            if len(split_list) == 4:
                start = int(split_list[0])
                end = int(split_list[1])
                name = split_list[2]
                if name not in geneName_dic.keys():
                    length = end-start
                    count = 1
                    temp = []
                    temp.append(count)
                    temp.append(length)
                    geneName_dic[name] = temp
                if name in geneName_dic.keys():
                    geneName_dic[name][0] += 1

    return geneName_dic


def read_all_file():
    dic_1 = {}
    i = 1
    while i < 23:
        address = make_address(i)
        name_dic = read_to_list(address)
        dic_1.update(name_dic)
```

```
        i+=1
    return dic_1

def gene_name_covert_to_uniprot(filename):
    a = open(filename)
    gene_name_dic = {}
    count = 0
    for line in a:
        count +=1
        if count >=2:
            line = line. strip()
            split_list = line.split()
            #print(split_list)
            if len(split_list) == 8:
                gene_name = split_list[2]
                uniprot_name = split_list[3]
                if gene_name not in gene_name_dic.keys():
                    uni_list = []
                    uni_list.append(uniprot_name)
                    gene_name_dic[gene_name] = uni_list
                if gene_name in gene_name_dic.keys():
                    old_list = gene_name_dic[gene_name]
                    old_list.append(uniprot_name)
                    new_list = list(set(old_list))
                    gene_name_dic[gene_name] = new_list
    return gene_name_dic

def find_uniprot_dnds(snp_dic, id_dic):
    uniprot_snp = {}
    for key, value in snp_dic.items():
        rate = value[0]/value[1]
        if key in id_dic:
            uniprot_list = id_dic[key]
            for item in uniprot_list:
                uniprot_snp[item] = rate
    return uniprot_snp

def writefile(dicname, writefilename):
    writefile = open(writefilename,'w')
    for keys, values in dicname.items():

        writefile.write(keys + '  ' + str(values))
        writefile.write('\n')


#put the snp count into a dictionary
def read_SNP_info(filename):
    readfile = open (filename)
    ProteinList = []
    for line in readfile:
        line = line.strip()
        split_list = line.split()
        protein_name = split_list[1]
        snp = split_list[3]
```

```
        temp = []
        temp.append(protein_name)
        temp.append(snp)
        ProteinList.append(temp)
    return ProteinList

def second_count_unique_snp(listname):
    AllList = {}
    snp_dic = {}
    for item in listname:
        protein_name = item[0]
        snp = item[1]
        snplist = []
        snplist.append(snp)
        if protein_name in snp_dic.keys():
            oldlist = snp_dic[protein_name]
            new = oldlist + snplist
            snp_dic[protein_name] = new
        else:
            snp_dic[protein_name] = snplist
    for key, value in snp_dic.items():
        count = len(set(value))
        AllList[key] = count
    return AllList

#---------------------------- Length Extraction ------------------------
-----------------

def third_read_length_info(filename):
    readfile = open (filename)
    ProteinList = []
    for line in readfile:
        line = line.strip()
        split_list = line.split()
        n = len(split_list)
        if n == 3:
            protein_name = split_list[0]
            length = int(split_list[1])
            temp = []
            temp.append(protein_name)
            temp.append(length)
            ProteinList.append(temp)
    return ProteinList

#because one uniprot ID could associated with more than one length, So I
will put all the length info in a uniprot ID dictionary, then use SNP
count divided by these length, then average the ratio to get the fincal
changing rate.

def uniprot_dic_unique_len(listname):
    d = {}
    for item in listname:
        protein_name = item[0]
        length = item[1]
```

```
        lenlist = []
        lenlist.append(length)
        if protein_name in d.keys():
            oldlist = d[protein_name]
            new = oldlist + lenlist
            d[protein_name] =new
        else:
            d[protein_name] = lenlist
    return d

#------------------------------ get changing rate -----------------------
--------------------
def caculate_changing_rate(snpdic, lendic):
    rate_dic = {}
    for key, value in lendic.items():
        if key in snpdic:
            len_array = np.array(value)
            snpcount = snpdic[key]
            r_array = snpcount/len_array
            average_r = r_array.mean()
            rate_dic[key] = average_r
    return rate_dic

def get_changing_rate_main(snpfile, lenfile):
    read_snp = read_SNP_info(snpfile)
    snpdic = second_count_unique_snp(read_snp)
    read_len = third_read_length_info(lenfile)
    lendic = uniprot_dic_unique_len(read_len)
    rate_dic = caculate_changing_rate(snpdic, lendic)
    return rate_dic

#-------------------------------- get x, y for plot ---------------------
--------------
def read_connectivity_file(connect_file):
    readfile_connect = open(connect_file)
    connect_dic = {}
    for line in readfile_connect:
        line = line. strip()
        split_list = line.split()
        protein = split_list[0]
        connect = int(split_list[1])
        connect_dic[protein] = connect
    return connect_dic

def get_x_y_plot(connect_dic, rate_dic):
    clist = []
    rlist = []
    for key, value in connect_dic.items():
        if key in rate_dic:
            r = rate_dic[key]
            clist.append(value)
            rlist.append(r)
    return clist, rlist
```

```
def denstify_plot(a, b):
    plt.style.use('fivethirtyeight')
    a = np.array(a)
    b = np.array(b)
    xy = np.vstack([a,b])
    z = gaussian_kde(xy)(xy)
    plt.rc('xtick', labelsize = 15)
    plt.rc('ytick', labelsize = 15)
    plt.scatter(a,b, c=z, s=10, edgecolor='')
    plt.xlabel('Protein Connectivity')
    plt.ylabel('Protein Polymorphism')

def density_change_scale(a, xlim, b, ylim):
    plt.style.use('fivethirtyeight')
    a = np.array(a)
    b = np.array(b)
    xy = np.vstack([a,b])
    z = gaussian_kde(xy)(xy)
    plt.rc('xtick', labelsize = 15)
    plt.rc('ytick', labelsize = 15)
    plt.scatter(a,b, c=z, s=10, edgecolor='')
    plt.xlim(0,120)
    plt.ylim(0,0.014)
    plt.xlabel('Protein Connectivity')
    plt.ylabel('Protein Polymorphism')

def threeDplot(a,b):
    p = mjp.mainj(10,10,a,b)
    x,y = mjp.get_meshgrid(10,10,a,b)
    plt.rc('xtick', labelsize = 12)
    plt.rc('ytick', labelsize = 12)
    fig = plt.figure()
    ax = fig.gca(projection='3d')
    surf = ax.plot_surface(x, y, p, cmap=cm.jet, rstride=1, cstride=1)
    ax.set_xlabel('Protein Connectivity', size =15)
    ax.set_ylabel('Protein Polymorphism', size = 15)

def check_connectivity_distribution(listname):
    con_array = np.array(listname)
    unique, counts = np.unique(con_array, return_counts=True)
    total = np.sum(counts)
    fre=counts/total
    return unique, fre

def connectivity_density_plot(a,b):
    plt.style.use('fivethirtyeight')
    a = np.array(a)
    b = np.array(b)
    xy = np.vstack([a,b])
    z = gaussian_kde(xy)(xy)
    plt.scatter(a,b, c=z, s=10, edgecolor='')
    plt.colorbar()
    plt.xlabel('Protein Connectivity')
    plt.ylabel('Frequency')
```

```
def main():
    snpfile = input("Please enter snp file: ")
    lenfile = input("Please enter length file: ")
    connect_file = input("Please enter connectivity file: ")
    rdic = get_changing_rate_main(snpfile, lenfile)
    cdic = read_connectivity_file(connect_file)
    x,y = get_x_y_plot(cdic, rdic)
    print('connectivity: lenth ',len(x), 'max ', max(x), 'min ', min(x),
'mean ', np.mean(x), 'median ', np.median(x))
    print('varibility: lenth ',len(y), 'max ', max(y), 'min ', min(y),
'mean ', np.mean(y), 'median ', np.median(y))
    return x,y




'''  modeling_join_probability.py    '''

import math
import numpy as np

def combine_end_2list(l1,l2):
    end_list = []
    length = len(l1)
    i = 0
    while i<length:
        temp = []
        con = l1[i]
        rate = l2[i]
        temp.append(con)
        temp.append(rate)
        end_list.append(temp)
        i+=1
    return end_list


def binvaule_convert(connect_bin, rate_bin, clist, rlist):
    cmax = max(clist)
    rmax = max(rlist)
    cmin = min(clist)
    rmin = min(rlist)
    connect_cap = abs(cmax-cmin)
    connect_interval = connect_cap/connect_bin
    rate_cap = abs(rmax-rmin)
    rate_interval = rate_cap/rate_bin
    c_binlist = []
    r_binlist = []
    for item in clist:
        connect_dif = item - cmin
        connect_category = math.ceil(connect_dif/connect_interval)
        if connect_category == 0:
```

```
            connect_category =1
        c_binlist.append(connect_category)
    for item in rlist:
        rate_dif = item  - rmin
        rate_category = math.ceil(rate_dif/rate_interval)
        if rate_category ==0:
            rate_category =1
        r_binlist.append(rate_category)

    return c_binlist, r_binlist

def join_frequent_count(arrayname, zeroarray):
    checklen = len(arrayname)
    for item in arrayname:
        first = item[0]-1
        second = item[1]-1
        zeroarray[first][second] +=1
    return zeroarray

def join_probability_array(join_count_array):
    array_total =np.sum(join_count_array)
    probability_array = join_count_array/array_total
    return probability_array

def join_probability(c_bin, r_bin):
    cbin_max = max(c_bin)
    rbin_max = max(r_bin)
    bin_list = combine_end_2list(c_bin, r_bin)
    pro_array = np.zeros((cbin_max, rbin_max))
    fre_count = join_frequent_count(bin_list, pro_array)
    join_pro = join_probability_array(fre_count)
    return join_pro

def mainj(cbin, vbin, clist, rlist):
    c_bin, r_bin = binvaule_convert(cbin, vbin, clist, rlist)
    p = join_probability(c_bin, r_bin)
    return p

def sign_jp_back_to_element(cbin, vbin, clist, rlist,p):
    c_bin, r_bin = binvaule_convert(cbin, vbin, clist, rlist)
    element_jp_val = []
    bin_list = combine_end_2list(c_bin, r_bin)
    for item in bin_list:
        b1 = item[0]-1
        b2 = item[1]-1
        element_jp_val.append(p[b1][b2])
    return element_jp_val

def get_meshgrid(connect_bin, rate_bin, clist, rlist):
    cmax = max(clist)
    rmax = max(rlist)
    cmin = min(clist)
    rmin = min(rlist)
    connect_cap = abs(cmax-cmin)
```

```
    connect_interval = connect_cap/connect_bin
    rate_cap = abs(rmax-rmin)
    rate_interval = rate_cap/rate_bin
    x = np.arange(cmin, cmax, connect_interval)
    y = np.arange(rmin, rmax, rate_interval)
    x, y = np.meshgrid(x, y)
    return x,y


''' modeling_c_v_indepently.py  '''


import numpy as np
import matplotlib.pyplot as plt
#model protein connectivity

def ini_net():
    a = np.array([0,1])
    b = np.fliplr([a])[0]
    c = np.vstack((a,b))
    return c
def prossible_connect(num):
    i =2
    while i < num:
        list(itertools.combinations([1,2,3], i))
        i+=1

#take node_number, network matrix--f1, and link matrix--check as input
def check_if_all_false(n, f, c):
    while (np.any(c)==False):
        random_p = np.random.rand(n)
        pro = np.sum(f,1)/np.sum(f)
        check = pro>random_p
        c = check
    return c

def build_network(nodes):
    f1 = ini_net()
    count = f1.shape[0]
    while count < nodes:
        node_number = f1.shape[0]
        random_p = np.random.rand(node_number)
        pro = np.sum(f1,1)/np.sum(f1)
        link = pro>random_p
        check = check_if_all_false(node_number, f1, link)
        newrow = np.vstack((f1,check))
        new_column = list(check)+([False])
        f1 = np.column_stack((newrow,new_column))
        count+=1
    return f1

#model protein SNPs with generation.
def model_snp_evalution(node_number):
    count = 1
```

```
    snp_list = np.array([])
    while count < node_number+1:
        rp = np.random.poisson(1, count)
        rp = np.array(rp)
        snp_list.resize(rp.shape)
        total = snp_list + rp
        snp_list = total.copy()
        count+=1
    return total

def model_snp_binomial(node_number):
    count = 1
    snp_list = np.array([])
    while count < node_number+1:
        rp = np.random.binomial(1, 0.1, count)
        rp = np.array(rp)
        snp_list.resize(rp.shape)
        total = snp_list + rp
        snp_list = total.copy()
        count+=1
    return total


'''   main_modeling.py '''

import numpy as np
import matplotlib.pyplot as plt
import modeling_c_v_indepently as mt
from scipy.stats import gaussian_kde
import modeling_join_probability as mjp
from mpl_toolkits.mplot3d import Axes3D
import sys
from matplotlib import cm
from scipy.stats import energy_distance
from scipy.stats import wasserstein_distance

#---------------------------------------------Binomial modeling ----------
---------------------------
def ini_net():
    a = np.array([0,1])
    b = np.fliplr([a])[0]
    c = np.vstack((a,b))
    return c

#take node_number, network matrix--f1, and link matrix--check as input
def check_if_all_false(n, f, c):
    while (np.any(c)==False):
        random_p = np.random.rand(n)
        pro = np.sum(f,1)/np.sum(f)
        check = pro>random_p
        c = check
    return c

def model_snp_exp_withtime_binomial(node, functions):
```

```
    f1 = ini_net()
    count = f1.shape[0]
    total_snplist = np.array([])
    k = 1
    '''k = 0.25, k = 0.1, k = 0.01, k = 2, k = 3, k = 0.5, k = 0.75, k =
-0.015, k = 0.015, k = 0.001, k = 0.0001'''

    e=2.718281
    c =[]
    while count < node:
        node_number = f1.shape[0]
        random_p = np.random.rand(node_number)
        pro = np.sum(f1,1)/np.sum(f1)
        link = pro>random_p
        check = check_if_all_false(node_number, f1, link)
        newrow = np.vstack((f1,check))
        new_column = list(check)+([False])
        f1 = np.column_stack((newrow,new_column))
        connect_count = np.array(f1.sum(axis=1))
        new_connect = (f1[count:])
        snplist = []

        if functions == 'exp':
            snplist.append(np.random.binomial(1, np.e**(-
k*connect_count)))

        # p cannot <0, otherwise there will be an error. because
pobability cannot less than 0.
        if functions == 'linear':
            ''' a = -1/80, a = -1/100, a = -1/1000, a = -0.0125, a = -
0.02, a = -0.015, a = -0.005, a = -0.008, a = -0.025, a = -0.03'''
            #a = -1/160
            a = -1/250
            b =1
            snplist.append(np.random.binomial(1,a*connect_count+b))
        if functions == 'sigmoid':
            p = 1/(1+np.e**(connect_count))
            snplist.append(np.random.binomial(1,p))
        if functions == 'gaussian':
            a =1
            c = 1/(np.pi*2)
            index = (connect_count**2)/(2*c)
            p = a*np.e**(-index)
            snplist.append(np.random.binomial(1,p))
        snparray = np.array(snplist)
        total_snplist.resize(snparray.shape)
        total = total_snplist + snparray
        total_snplist = total.copy()
        count+=1
    return connect_count, total_snplist

#----------------------------------------poisson modeling----------------
--------------------
```

```
def model_snp_exp_withtime_possion(node, functions):
    f1 = ini_net()
    count = f1.shape[0]
    total_snplist = np.array([])
    k = 1
    e=2.718281
    c =[]
    while count < node:
        node_number = f1.shape[0]
        random_p = np.random.rand(node_number)
        pro = np.sum(f1,1)/np.sum(f1)
        link = pro>random_p
        check = check_if_all_false(node_number, f1, link)
        newrow = np.vstack((f1,check))
        new_column = list(check)+([False])
        f1 = np.column_stack((newrow,new_column))
        connect_count = np.array(f1.sum(axis=1))
        new_connect = (f1[count:])
        snplist = []
        if functions == 'exp':
            snplist.append(np.random.poisson(np.e**(-k*connect_count)))
        if functions == 'linear':
            a = -1/80
            b =1
            snplist.append(np.random.poisson(a*connect_count+b))
        if functions == 'sigmoid':
            p = 1/(1+np.e**(connect_count))
            snplist.append(np.random.poisson(p))
        if functions == 'gaussian':
            a =1
            c = 1/(np.pi*2)
            index = (connect_count**2)/(2*c)
            p = a*np.e**(-index)
            snplist.append(np.random.poisson(p))

        snparray = np.array(snplist)
        total_snplist.resize(snparray.shape)
        total = total_snplist + snparray
        total_snplist = total.copy()
        count+=1
    return connect_count, total_snplist

#---------------------------------modeling length -----------------------
------

def main_len_100_times_snp(node, func, distribution_func):
    if distribution_func == 'poisson':
        a,b = model_snp_exp_withtime_possion(node, func)
        b = np.reshape(b, node)
        l = max(b)
        l = 100*l
        b = b/l
    if distribution_func == 'binomial':
        a,b = model_snp_exp_withtime_binomial(node, func)
```

```
        b = np.reshape(b, node)
        l = max(b)
        l = 100*l
        b = b/l

    #print('connectivity: lenth ',len(a), 'max ', max(a), 'min ', min(a),
'mean ', np.mean(a), 'median ', np.median(a))
    #print('varibility: lenth ',len(b), 'max ', max(b), 'min ', min(b),
'mean ', np.mean(b), 'median ', np.median(b))
    return a, b

#-----------------------------------plot figure-----------------------------
------

def density_plot(a, b):
    plt.style.use('fivethirtyeight')
    xy = np.vstack([a,b])
    z = gaussian_kde(xy)(xy)
    plt.scatter(a,b, c=z, s=10, edgecolor='')
    plt.colorbar()
    plt.xlabel('Protein Connectivity')
    plt.ylabel('Protein Variability')

def surface_plot(a, b):
    p = mjp.mainj(10,10,a,b)
    x,y = mjp.get_meshgrid(10,10,a,b)
    plt.rc('xtick', labelsize = 8)
    plt.rc('ytick', labelsize = 8)
    fig = plt.figure()
    ax = fig.gca(projection='3d')
    surf = ax.plot_surface(x, y, p, cmap=cm.jet, rstride=1, cstride=1)
    fig.colorbar(surf, shrink=0.5, aspect=20)
    ax.set_xlabel('Protein Connectivity')
    ax.set_zlabel('Join Probability of connectivity / variability')
    ax.set_ylabel('Protein Variability')

#----------------------------------Test-------------------------------------
------

def test_code_snp_function(numoftest,node, func, distritution_func):
    i = 0
    con_max_list = []
    con_min_list = []
    con_mean_list = []
    con_median_list = []
    snp_max_list = []
    snp_min_list = []
    snp_mean_list = []
    snp_median_list = []
    each_run_connect_list =[]
    each_run_snp_list = []
    all_con_list = []
    all_snp_list = []
```

```
    while i < numoftest:
        connect, snp =
main_len_100_times_snp(node,func,distritution_func)
        #----------------------------------------------------------
        max_connect = max(connect)
        min_connect = min(connect)
        mean_connect = np.mean(connect)
        median_connect = np.median(connect)

        max_snp = max(snp)
        min_snp = min(snp)
        mean_snp = np.mean(snp)
        median_snp = np.median(snp)
        #-------------------------------------------------------
        con_max_list.append(max_connect)
        con_min_list.append(min_connect)
        con_mean_list.append(mean_connect)
        con_median_list.append(median_connect)

        snp_max_list.append(max_snp)
        snp_min_list.append(min_snp)
        snp_mean_list.append(mean_snp)
        snp_median_list.append(median_snp)
        #-------------------------------------------------
        each_run_connect_list.append(max_connect)
        each_run_connect_list.append(min_connect)
        each_run_connect_list.append(mean_connect)
        each_run_connect_list.append(median_connect)

        each_run_snp_list.append(max_snp)
        each_run_snp_list.append(min_snp)
        each_run_snp_list.append(mean_snp)
        each_run_snp_list.append(median_snp)

        all_con_list.append(each_run_connect_list)
        all_snp_list.append(each_run_snp_list)
        print(i)
        i+=1
    all_con_list = np.array(all_con_list)
    all_snp_list = np.array(all_snp_list)
    #return 10 items
    return con_max_list, con_min_list, con_mean_list, con_median_list,
snp_max_list, snp_min_list, snp_mean_list, snp_median_list, all_con_list,
all_snp_list

def uncoupled_modeling(node_number):
     import modeling_c_v_indepently as mt
     a = mt.build_network(node_number)
     a = a.sum(axis = 1)
     b = mt.model_snp_evalution(node_number)
     b = np.reshape(b,node_number)
     l = max(b)
     l = 100*l
     b = b/l
```

```
     return a, b

#-----------------------------------------------------------------------------
--------------------

'''
2018-06-06
To solve the question: what is the highest possible value (approximately)
of protein connectivity in simulations? so i wrote the method to run 100
simulation of finding the list of max of connectivity.
'''
def simulate_100times(nodes, func, num):
    highest_connectivity_list = []
    i = 0
    while i < num:
        x,y = model_snp_exp_withtime_binomial(nodes,func)
        highest_connectivity_list.append(max(x))
        i+=1
    res = np.array(highest_connectivity_list)
    return res

#-----------------------------------------------------------------------------
-------------------
'''try different parameter of a and b in linear v-c function, add the
condition in the while loop, when the result is out of the range.
'''
def model_snp_exp_withtime_binomial(node, functions, m, n):
    f1 = ini_net()
    count = f1.shape[0]
    total_snplist = np.array([])
    while count < node:
        node_number = f1.shape[0]
        test = f1.sum(axis=1)
        if np.any(test>99) == True:
            #print('connectivty max = ', max(test))
            break
        random_p = np.random.rand(node_number)
        pro = np.sum(f1,1)/np.sum(f1)
        link = pro>random_p
        check = check_if_all_false(node_number, f1, link)
        newrow = np.vstack((f1,check))
        new_column = list(check)+([False])
        f1 = np.column_stack((newrow,new_column))
        connect_count = np.array(f1.sum(axis=1))
        new_connect = (f1[count:])
        snplist = []
# p cannot <0, otherwise there will be an error. because pobability
cannot less than 0.
        if functions == 'linear':
            ''' a = -1/80, a = -1/100, a = -1/1000, a = -0.0125, a = -
0.02, a = -0.015, a = -0.00
5, a = -0.008, a = -0.025, a = -0.03'''
            a = m
            b = n
```

```
            res = a*connect_count+b
            res = np.where(res>1, 1, res)
            res = np.where(res<0, 0, res)
            snplist.append(np.random.binomial(1,res))

        snparray = np.array(snplist)
        total_snplist.resize(snparray.shape)
        total = total_snplist + snparray
        total_snplist = total.copy()
        count+=1
    return connect_count, total_snplist


#-------------------------------------Normalize to the same scale---------
-------------------

def normalization_c_v(model_connect, model_rate, real_connect,
real_rate):
    real_c_max = max(real_connect)
    real_r_max = max(real_rate)
    model_c_max = max(model_connect)
    model_r_max = max(model_rate)
    normal_real_c = real_connect/real_c_max
    normal_real_r = real_rate/real_r_max
    normal_model_c = model_connect/model_c_max
    normal_model_r = model_rate/model_r_max
    return normal_model_c, normal_model_r, normal_real_c, normal_real_r

#-----------------------------Validation with energy distant and EMD------
--------------------

def two_validate(a, b, c, d):
    emd = wasserstein_distance(a, b, c, d)
    energy = energy_distance(a, b, d, c)
    #print('EMD value is: ', emd)
    #print('Energy value is: ', energy)
    return emd, energy


def write_list_to_file(listname, writefilename):
    w = open(writefilename,'w')
    for item in listname:
        w.write(str(item))
        w.write('\n')

if __name__=="__main__":
    a,b = main_len_100_times_snp(1000,'exp','binomial')
    write_list_to_file(a,'connect_file_write.txt')
    write_list_to_file(b, 'change_rate_file_write.txt')



''' Validation_test.py'''
```

```
import numpy as np
import itertools
from scipy.spatial.distance import pdist


def energyStatistic(X,Y,k=2):
    n1=len(X)
    n2=len(Y)
    ik=1/k
    a=eucAlld(X,X,k,ik)
    b=eucAlld(X,Y,k,ik)
    c=eucAlld(Y,Y,k,ik)

    return (n1*n2/(n1+n2))*((2/(n1*n2))*b-(1/n1**2)*a-(1/n2**2)*c)


def energyDistance(X,Y):
    n1=len(X)
    n2=len(Y)
    a=np.mean(cdist(X,X))
    b=np.mean(cdist(X,Y))
    c=np.mean(cdist(Y,Y))
    return 2*b-a-c


from scipy.optimize import linear_sum_assignment as lsa
import numpy as np

samples = np.random.multivariate_normal([0,0],np.eye(2),size=1000)


def prep_data(samples):
    h,x,y = np.histogram2d(samples[:,0],samples[:,1],bins=10)
    center_x = x[:-1] + 0.5*( x[1:] - x[:-1] )
    center_y = y[:-1] + 0.5*( y[1:] - y[:-1] )
    count_ind = np.vstack( np.where(h > 0) ).T
    xyz = np.array([ [x[i[0]],y[i[1]],h[i[0],i[1]]] for i in count_ind])
    return xyz


def wmetric(x, x_weight, y, y_weight):
    ind_x=np.where(x_weight>1)[0]
    a=x.reshape(x.shape[0],2).tolist()
    ind_y=np.where(y_weight>1)[0]
    b=y.reshape(y.shape[0],2).tolist()

    for i in ind_x:
        for j in range(int(x_weight[i])-1): a.insert(0,x[i,:].tolist())
    for i in ind_y:
        for j in range(int(y_weight[i])-1): b.insert(0,y[i,:].tolist())

    W=np.array([[np.sqrt((xx[0]-yy[0])**2+(xx[1]-yy[1])**2) for xx in a]
for yy in b])
    return sum([W[i[0],i[1]] for i in np.vstack(lsa(W)).T])
```

```
def computeDistance(X,Y):
    a=prep_data(X)
    b=prep_data(Y)
    D=wmetric(a[:,:2],a[:,2],b[:,:2],b[:,2])
    return D


import numpy as np
import energyStat as es
import main_modeling as mm
import matplotlib.pyplot as plt
import connectivity_SNPrate_plot as csp


#snp_file = 'unip_dS_less_than_one_8_20_18_high_confi.txt'
#snp_file = 'unip_dn_less_ten_8_20_18_high_confidence.txt'
#snp_file = 'ensemble_snp_4_13_first_try.txt'
#connect_file = 'STRING_binding_connectivity_8_25_18.txt'

def valiation_new_sergio_code(connect_file, snp_file):
    c, d = csp.get_dnds_connect_list(connect_file, snp_file)

    a_exp, b_exp = mm.main_len_100_times_snp(1000,'exp','binomial')
    a_lin, b_lin = mm.main_len_100_times_snp(1000,'linear','binomial')
    a_unc, b_unc = mm.uncoupled_modeling(1000)

    a1, b1, c1, d1 = mm.normalization_c_v(a_exp, b_exp, c, d)
    a2, b2, c2, d2 = mm.normalization_c_v(a_lin, b_lin, c, d)
    a3, b3, c3, d3 = mm.normalization_c_v(a_unc, b_unc, c, d)

    X1 = np.array((a1, b1)).T
    Y1 = np.array((c1, d1)).T
    X2 = np.array((a2, b2)).T
    Y2 = np.array((c2, d2)).T
    X3 = np.array((a3, b3)).T
    Y3 = np.array((c3, d3)).T

    ed_e = es.energyDistance(X1,Y1)
    #ed_e = es.energyStatistic(X1,Y1)
    print('exp distance: ', ed_e)
    ed_l =  es.energyDistance(X2,Y2)
    #ed_l =  es.energyStatistic(X2,Y2)
    print('linear distance: ', ed_l)
    ed_c = es.energyDistance(X3,Y3)
    #ed_c = es.energyStatistic(X3,Y3)
    print('uncouple distance: ', ed_c)


def linear_validate(a, b):

    connect_file = 'STRING_binding_connectivity_8_25_18.txt'
    #connect_file = 'nei_connectivity.txt'
    #connect_file = 'dir_connectivity.txt'
```

```
    snp_file = 'ensemble_snp_4_13_first_try.txt'
    c, d = csp.get_dnds_connect_list(connect_file, snp_file)
    a_lin, b_lin = main_len_100_times_snp(1000,'linear','binomial', a, b)
    a2, b2, c2, d2 = normalization_c_v(a_lin, b_lin, c, d)
    X2 = np.array((a2, b2)).T
    Y2 = np.array((c2, d2)).T
    ed_l =  es.energyStatistic(X2,Y2)
    print('a = ', a, 'b = ',b, ' Linear Distance: ', ed_l)
    return ed_l

def main():
    for j in range (40,51):
        b = j/10
        for i in range(30,40):
            a = -i/100
            #print(a,b)
            res = linear_validate(a, b)
```
