# Supplementary material for: Dependency Between Protein–Protein Interactions and Protein Variability and Evolutionary Rates in Vertebrates: Observed Relationships and Stochastic Modeling
Source: J Mol Evol. 2019 Jul 13;87(4):184–98. doi: 10.1007/s00239-019-09899-z (PMC6658588; doi:10.1007/s00239-019-09899-z)
Supplement: Supplementary file 14 — Supplementary material 14 (PDF 36 kb). Supplementary Material 2. Annotation for the data files [file 239_2019_9899_MOESM14_ESM.pdf]

The Part1_data_file.txt and Part2_data_file contains information used for the data anaylsis in this paper. You can use the index number to find the data file accordingly:

-------------------------Part1_data_file ------------------------------

1 STRING Connectivity

16791 Direct Complex Connectivity

18898 Indirect Complex Connectivity

21394 Reaction Complex Connectivity

24893 Neighboring Reaction Connectivity

28663 Agile Connectivity

57429 Polymorphism Changing Rate

119037 uniprot_ratio_high_confidence_2019

177485 uniprot_dn_high_confi_2019

235954 uniprot_dS_high_confi_2019

294419 chicken_uniprot_connect_8_27_18

295333 gallus_gallus_variation

535958 chicken_len

568937 pig_uniprot_connect_8_27_18

570141 sus_scrofa_variation

706853 pig_len

------------------------Part2_data_file-----------------------------

2 mouse_uniprot_connect_8_27_18

18023 mus_musculus_variation

290548 mouse_len

426471 zebrafish_uniprot_connect_8_27_18

430954 danio_rerio_variation.txt

556739 Zebrafish_len.txt

607773 mouse_uniprot_dDdS_ratio_2019

646188 mouse_dN_only_2019

684602 mouse_dS_only_2019
