# Supplementary figures and images for: Dependency Between Protein–Protein Interactions and Protein Variability and Evolutionary Rates in Vertebrates: Observed Relationships and Stochastic Modeling
Source: J Mol Evol. 2019 Jul 13;87(4):184–98. doi: 10.1007/s00239-019-09899-z (PMC6658588; doi:10.1007/s00239-019-09899-z)

$a = -0.001, b = 1$

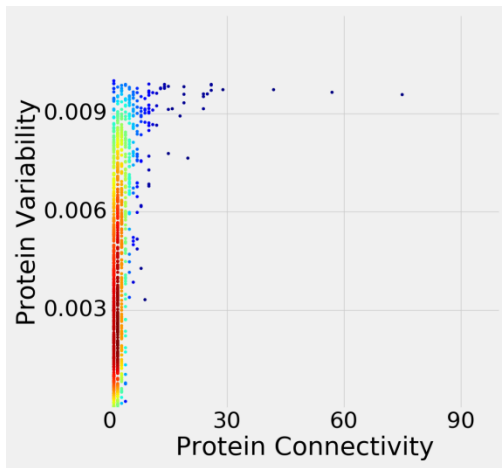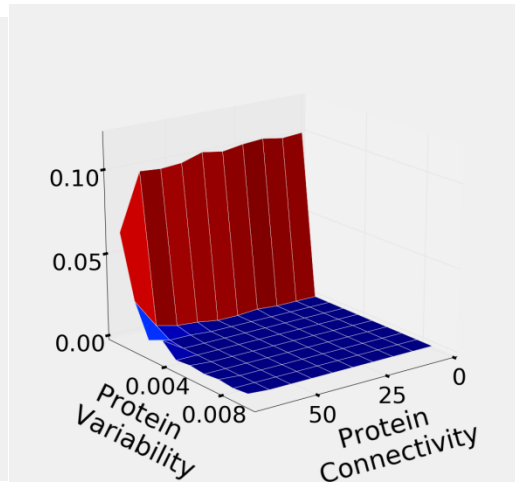

$a = -0.002, b = 0.2$

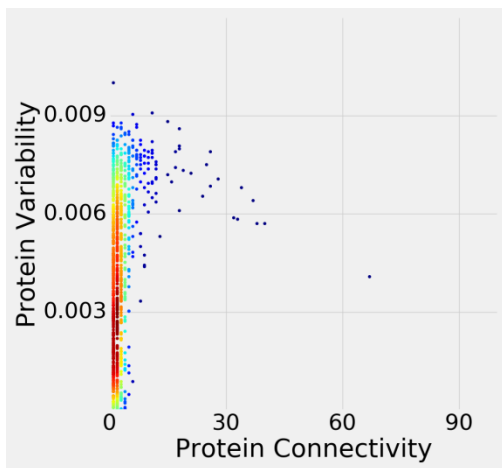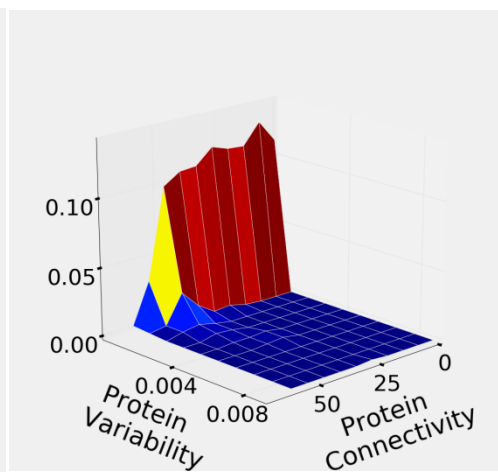

$a = -0.003, b = 0.3$

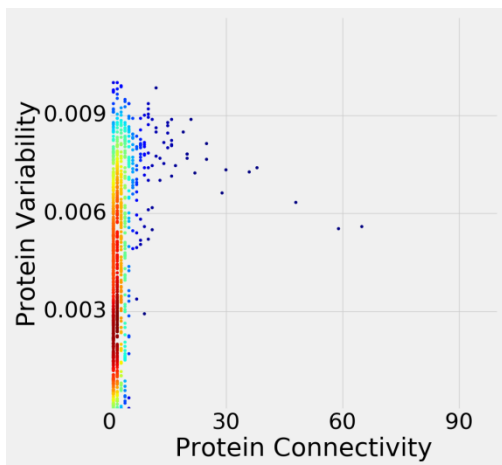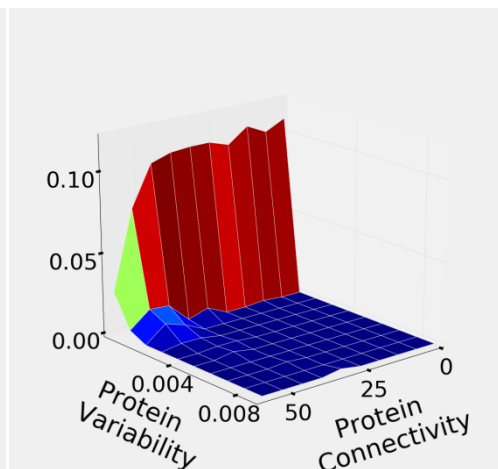

$a = -0.004, b = 0.4$

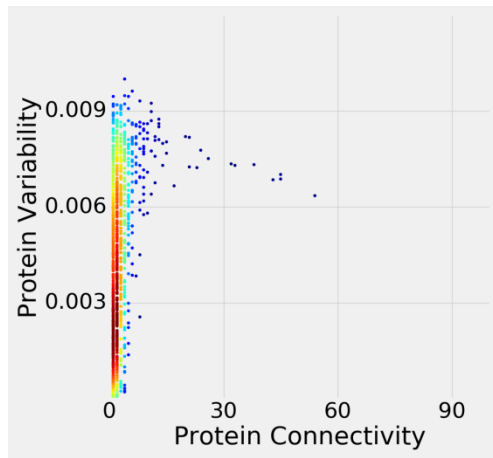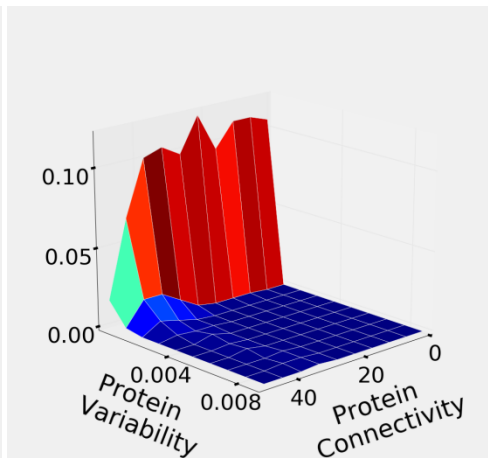

$a = -0.005, b = 0.5$

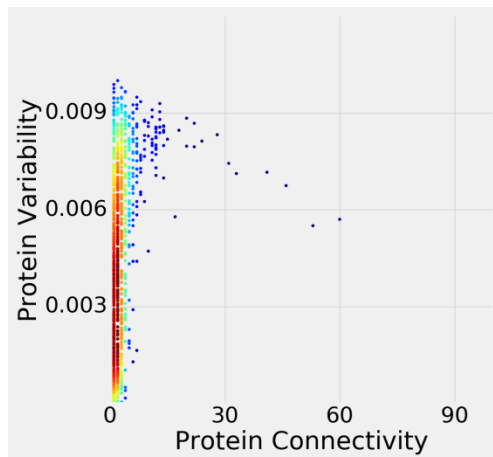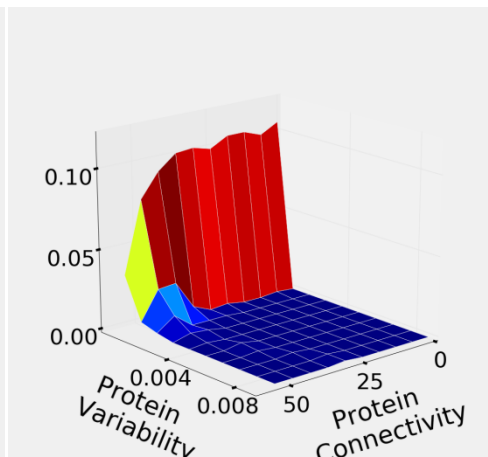

$a = -0.006, b = 0.6$

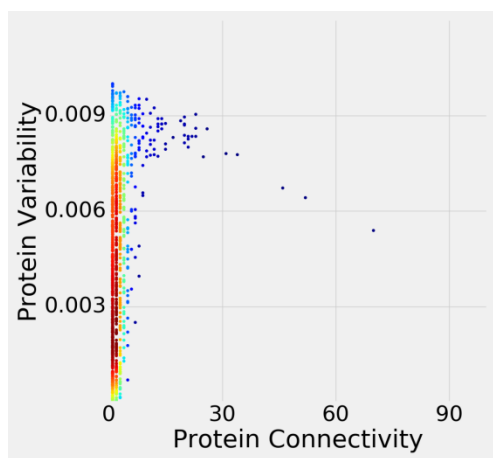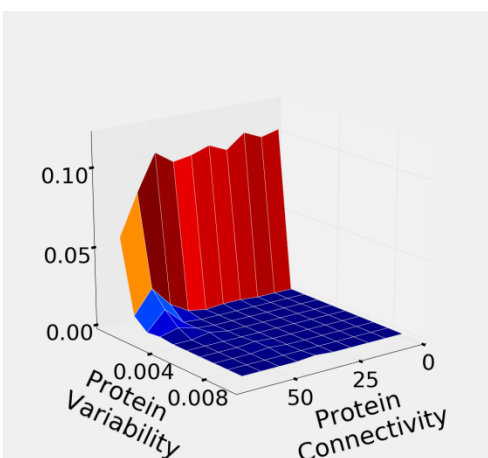

$a = -0.007, b = 0.7$

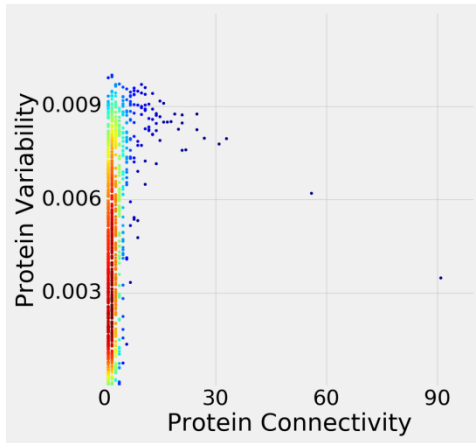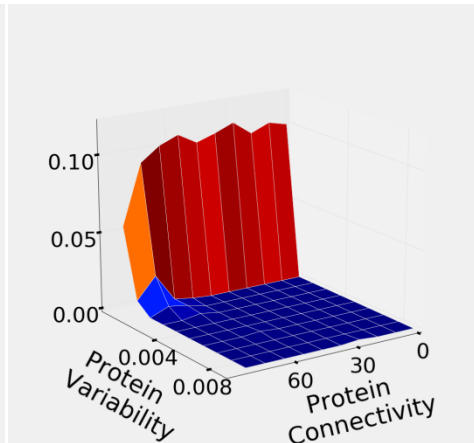

$a = -0.008, b = 0.8$

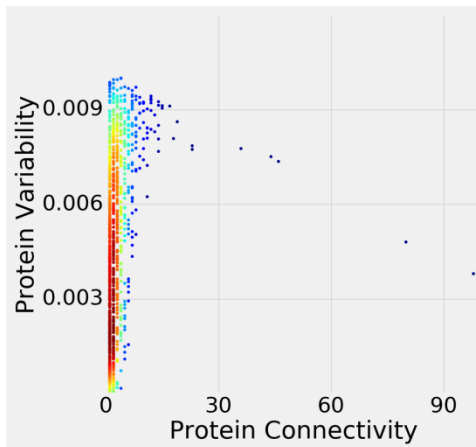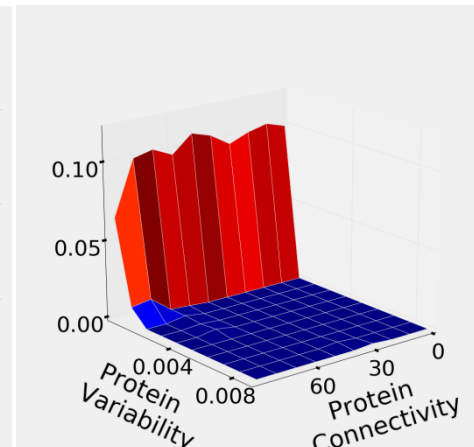

$a = -0.009, b = 0.9$

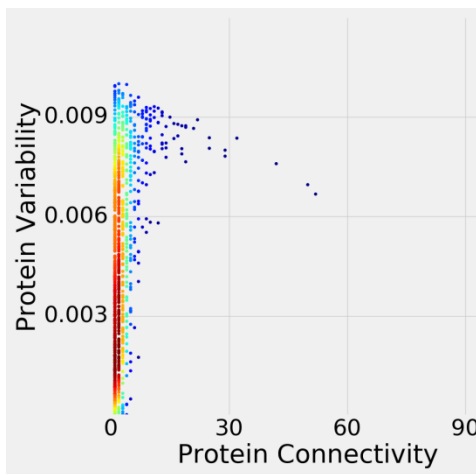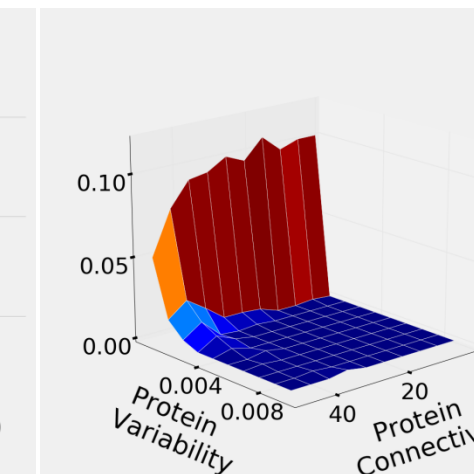

$a = -0.01, b = 1$

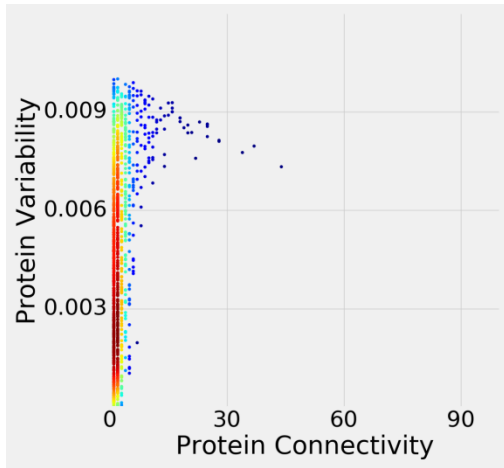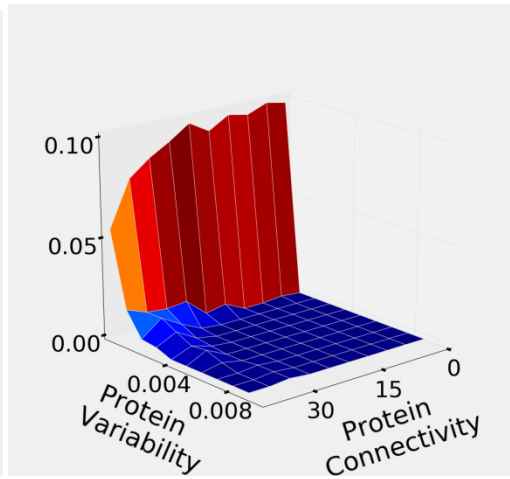

$a = -0.0125, b = 1$

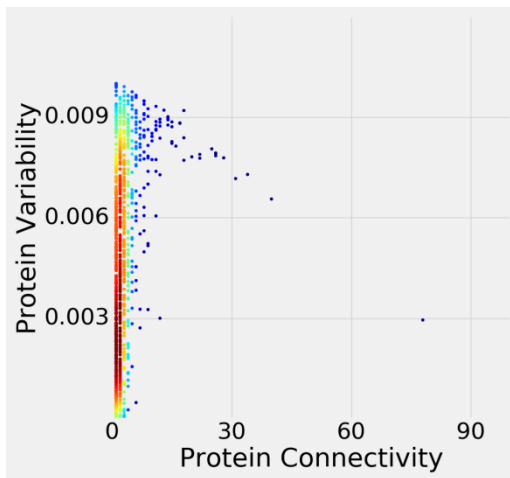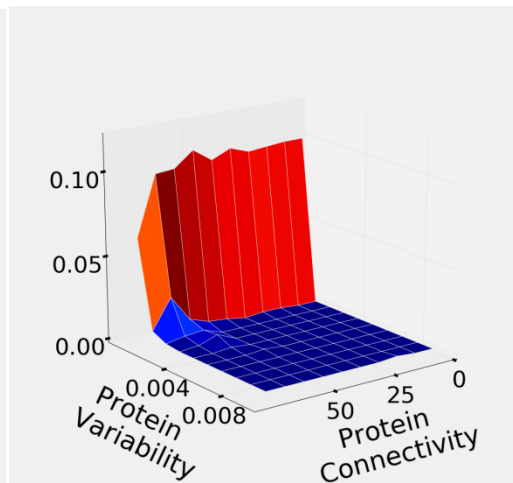

$a = -0.015, b = 1$

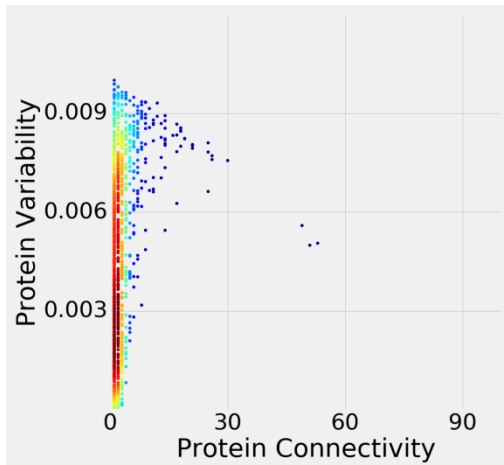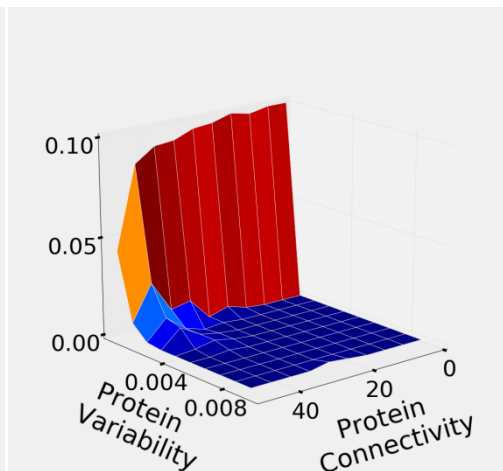

$a = -0.004, b = 1$

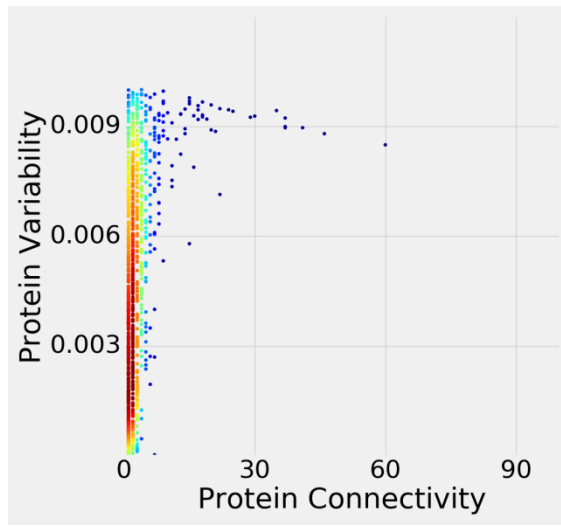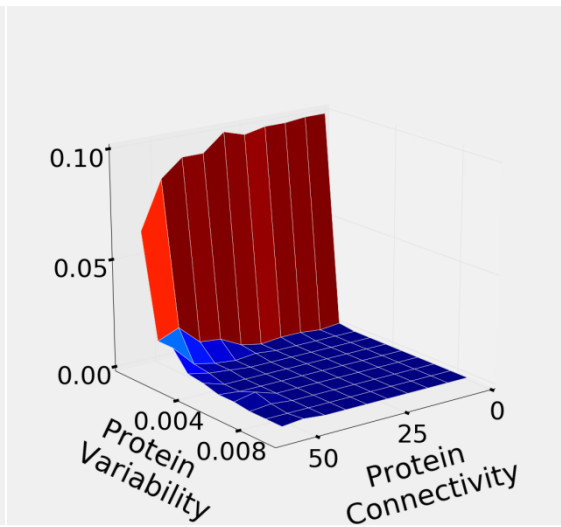

$a = -0.009, b = 1$

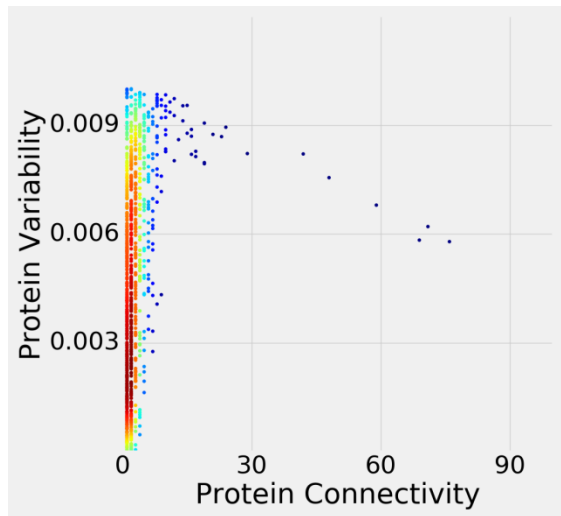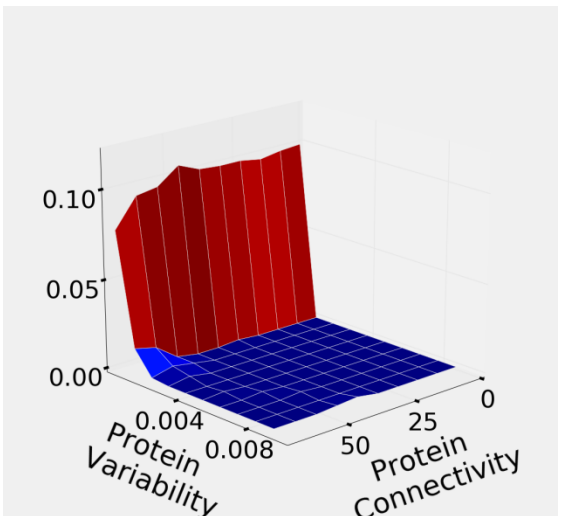

Supplement: Supplementary file 17 — Supplementary material 17 (PDF 1795 kb). Supplementary Material 5. Simulation results (negative linear V-C function). Density plots (left panes) and 3D surface plots (right panes) of simulated protein variability vs. protein connectivity, with protein variability and connectivity linked via negative linear function (second modeling scenario). Intercept and slope parameter values are shown next to each simulation plot [file 239_2019_9899_MOESM17_ESM.pdf]

$K=0.0001$

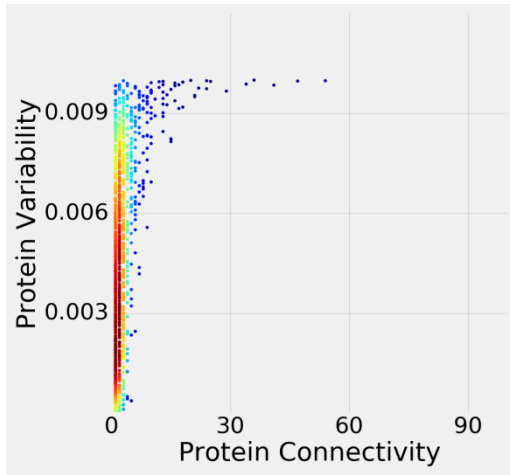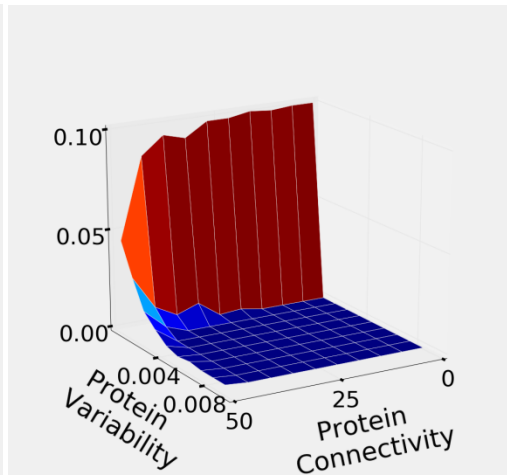

$K=0.001$

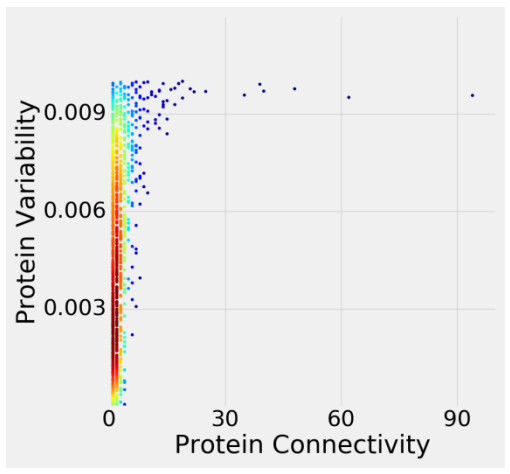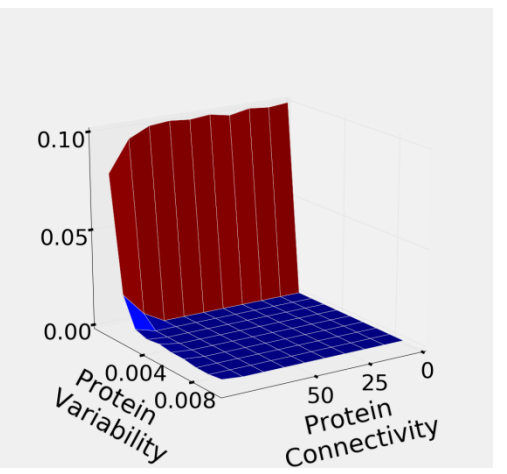

$K = 0.01$

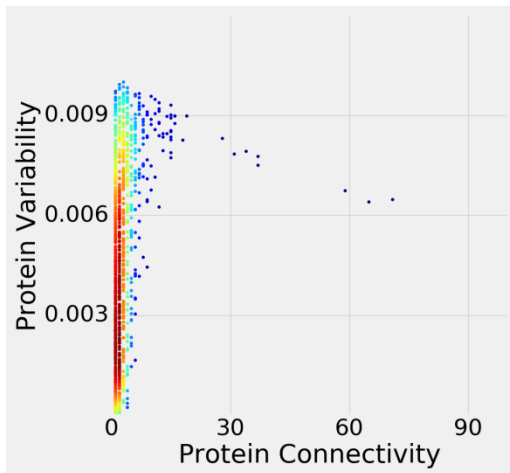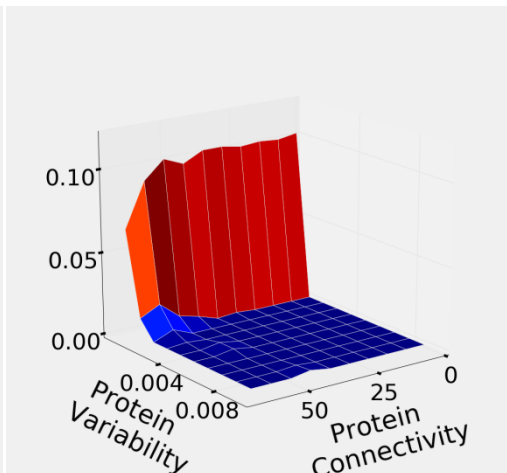

$K = 0.1$

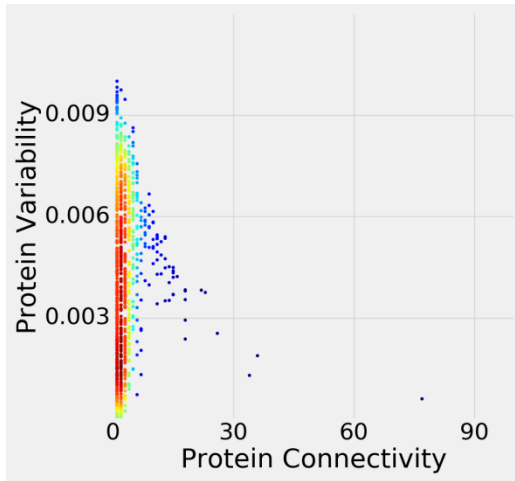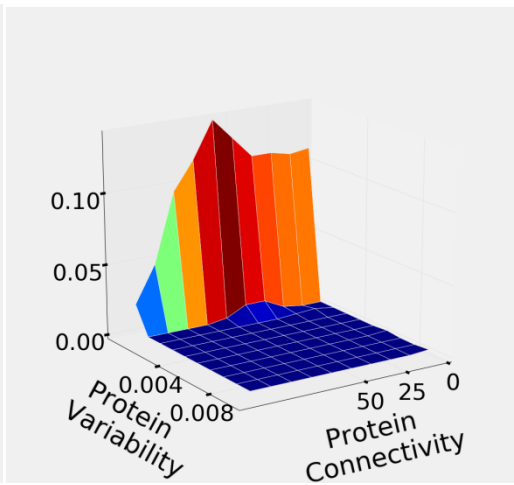

$K = 0.5$

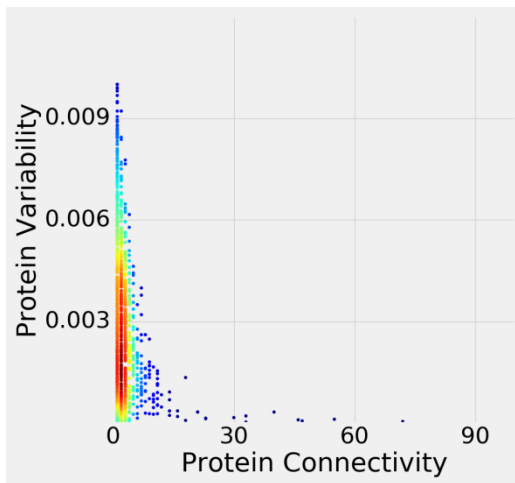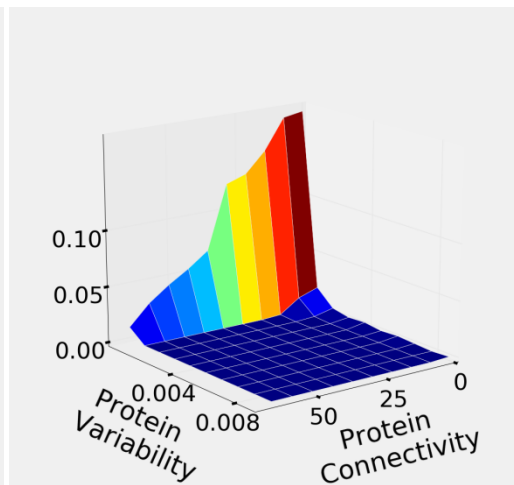

$K = 0.9$

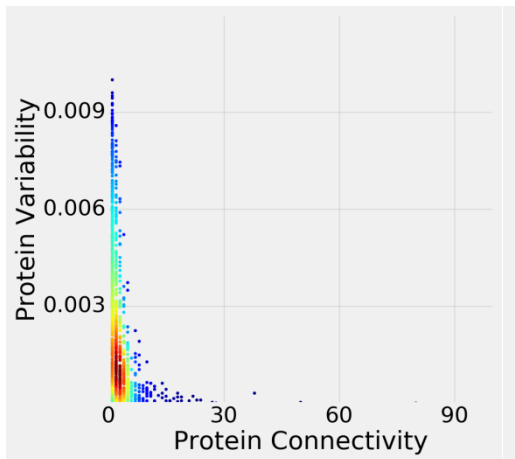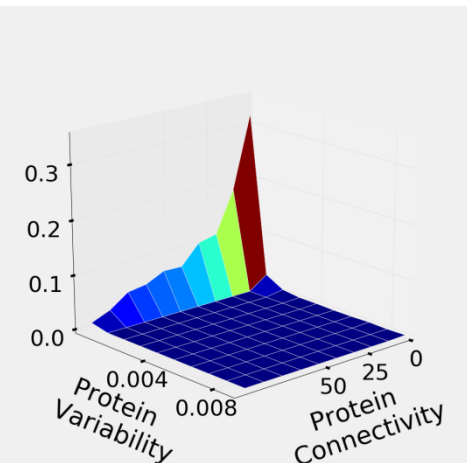

$K = 1$

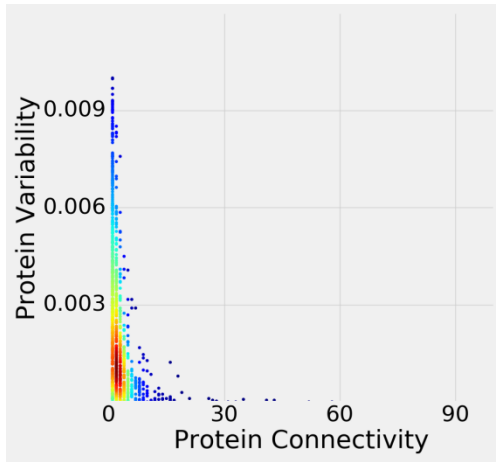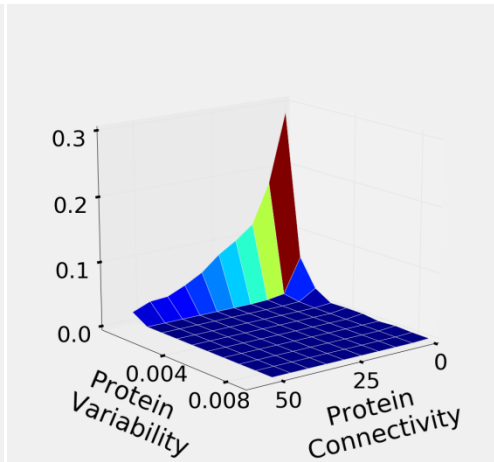

$K = 2$

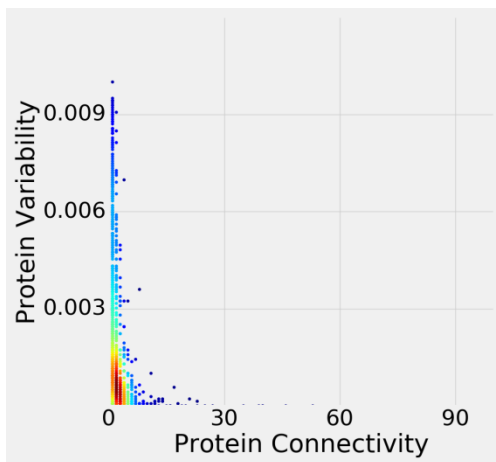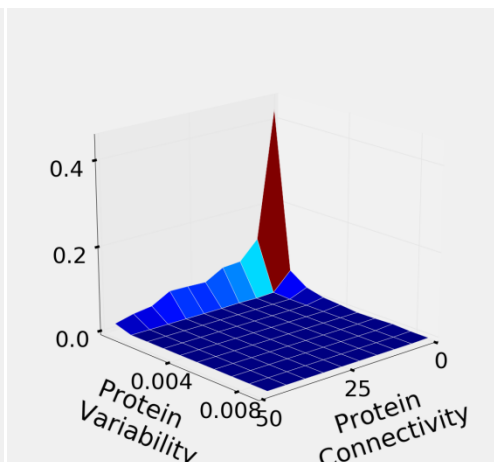

$K = 3$

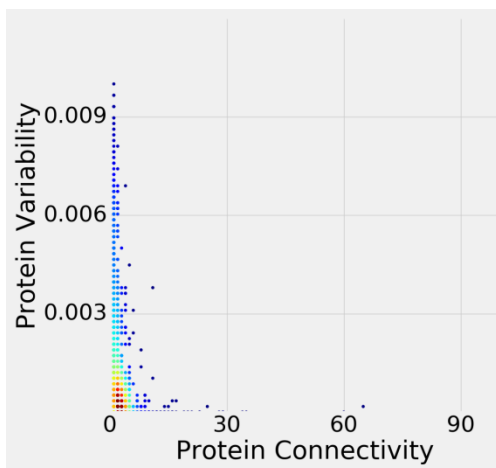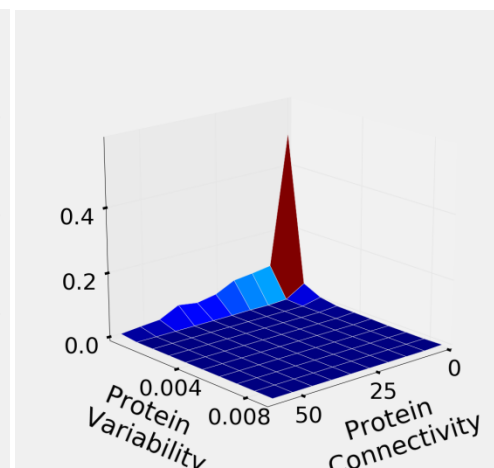

K = 4

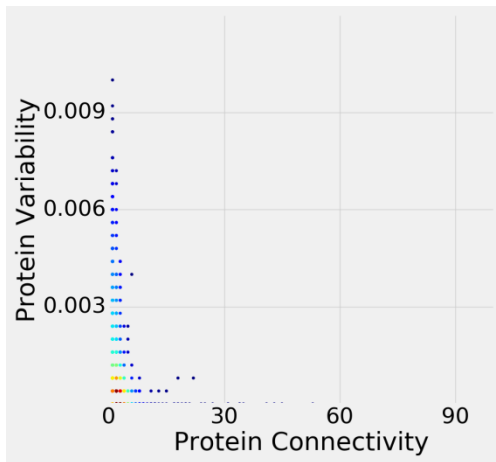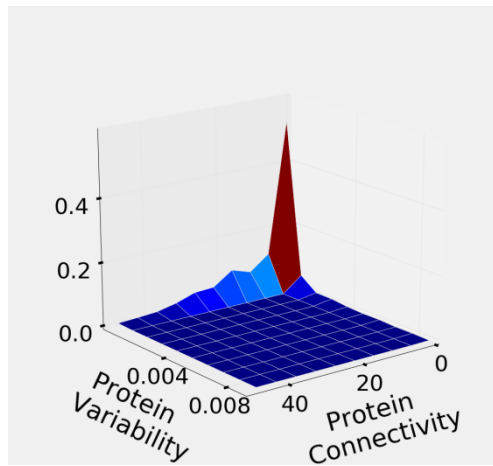

K = 5

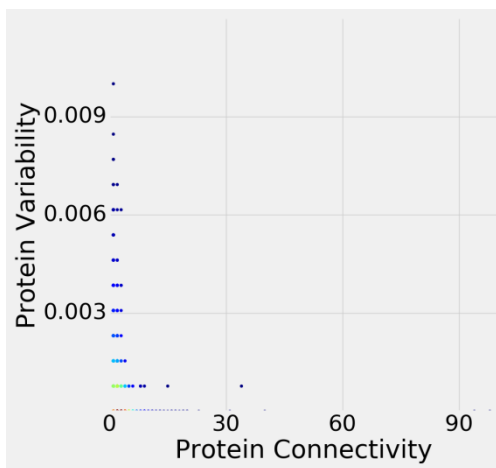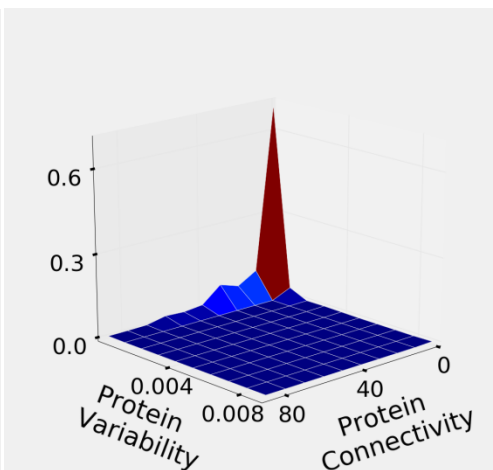

K = 6

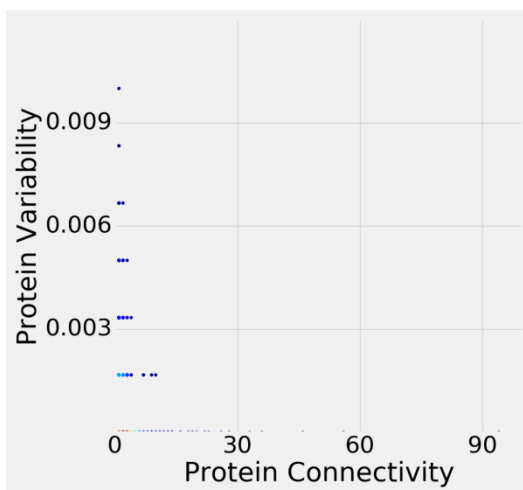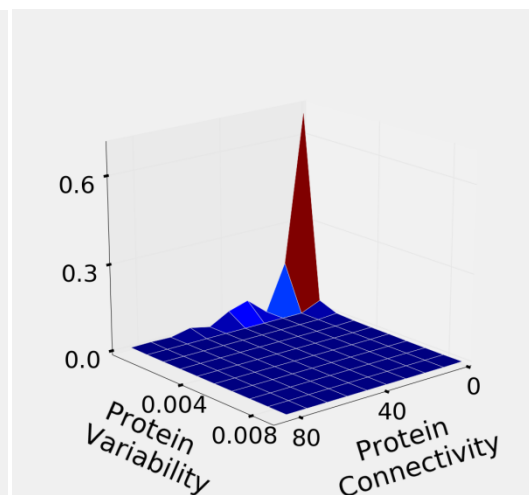

Supplement: Supplementary file 18 — Supplementary material 18 (PDF 1341 kb). Supplementary Material 6. Simulation results (exponential decay V-C function). Density plots (left panes) and 3D surface plots (right panes) of simulated protein variability vs. protein connectivity, with protein variability and connectivity linked via exponential decay function (third modeling scenario). Exponential decay constant parameter value is shown next to each simulation plot [file 239_2019_9899_MOESM18_ESM.pdf]
