# Supplementary material for: Dependency Between Protein–Protein Interactions and Protein Variability and Evolutionary Rates in Vertebrates: Observed Relationships and Stochastic Modeling
Source: J Mol Evol. 2019 Jul 13;87(4):184–98. doi: 10.1007/s00239-019-09899-z (PMC6658588; doi:10.1007/s00239-019-09899-z)
Supplement: Supplementary file 19 — Supplementary material 19 (PDF 86 kb). Supplementary Material 7. ED measure (distributional distance) between the simulated protein variability vs. protein connectivity distribution (with protein variability and connectivity linked via negative linear function, second modeling scenario) and observed human data from STRING database, shown across a wide range of negative linear function parameters (a and b), averaged over 100 simulations for each parameter combination. Simulations were carried out for 15903 nodes, to match exactly with the human STRING data. Darker orange color indicates higher ED values (more distributional divergence), less saturated yellow color --- lower ED values [file 239_2019_9899_MOESM19_ESM.pdf]

ED Statistic (average of 100 simulations)

	STRING Connectivity
	Ensembl Intraspecific Variability
No. of nodes	15903 nodes
V-C function	Negative linear

a\b	0.1	0.2	0.3	0.4	0.5	0.6	0.7	0.8	0.9	1
-0.001	482.8473	510.8554	532.2574	546.0485	548.6243	561.8009	572.1691	561.7317	574.1527	573.2847
-0.002	463.105	517.6218	531.3267	553.7839	558.992	556.6982	565.911	573.3954	574.0804	573.3179
-0.003	469.3331	512.078	532.2618	540.7438	561.6807	568.1738	566.7659	561.7564	574.6854	569.1856
-0.004	469.8749	501.2316	531.3268	543.0242	552.6226	568.4141	569.5496	571.273	569.6573	584.0687
-0.005	453.5477	512.2815	528.4564	545.0272	550.4163	560.1715	563.5599	573.9902	573.7059	581.977
-0.006	452.4594	514.4723	536.9141	549.2353	548.3923	557.6353	570.4002	573.7463	571.389	575.7604
-0.007	433.1572	500.408	528.0354	546.1468	548.3181	555.7767	554.447	569.0153	581.2105	577.4023
-0.008	427.9625	502.9432	523.8229	531.59	553.0468	558.9377	563.0642	568.5458	574.808	571.7868
-0.009	420.3298	494.9144	523.9415	528.4102	553.1965	557.1373	565.65	558.6625	574.4976	580.0507

a\b	1	1.1	1.2	1.3	1.4	1.5	1.6	1.7	1.8	1.9	2
-0.01	572.5145	575.2718	582.2129	586.2708	575.8508	579.726	566.9318	582.8855	570.7961	581.8529	578.3423
-0.02	563.8761	562.9378	578.7083	589.969	581.5466	576.3596	581.0706	584.0614	577.2548	576.4192	588.5815
-0.03	555.8343	563.7309	576.0519	579.442	578.0039	578.7277	585.6258	580.3646	573.427	585.7233	583.1916
-0.04	542.3271	551.4577	569.2363	570.4882	570.2898	580.1845	580.2257	572.7289	579.3421	584.3442	583.2299
-0.05	532.92	548.1851	572.4747	568.95	569.4934	567.2494	578.3341	586.7585	577.8731	575.2725	582.7763
-0.06	518.4003	541.7201	559.1709	564.4132	569.9826	569.566	572.1053	575.4223	576.7198	578.5971	581.7625
-0.07	508.7358	524.2917	546.9495	562.2721	574.7973	573.3204	567.7807	572.2885	569.4473	570.5098	560.7642
-0.08	496.3959	516.3893	544.2453	554.4062	572.628	569.6687	565.6496	565.225	565.77	569.5208	564.0762
-0.09	481.0172	492.4934	525.976	540.3375	555.4216	560.5307	562.0402	567.9576	563.2984	562.6755	571.6605

a\b	2	2.1	2.2	2.3	2.4	2.5	2.6	2.7	2.8	2.9	3
-0.1	579.6575	568.4462	581.5057	581.6499	576.3149	568.797	573.2817	583.0418	576.6164	586.6613	577.883
-0.11	565.26	574.125	567.8107	574.2093	561.1201	563.907	571.5025	569.4275	574.1516	577.9431	562.3569
-0.12	561.4913	570.2638	575.6106	563.9342	573.0904	568.2099	575.4456	574.3402	577.411	570.2611	573.0874
-0.13	563.8542	563.8084	568.3533	571.5757	571.0415	569.0555	575.796	561.8901	575.7674	574.3089	573.2327
-0.14	561.3017	569.1982	566.5602	569.3019	564.9139	568.2653	565.8882	568.9153	567.8577	569.2955	560.809
-0.15	559.6746	557.2055	567.5673	562.9141	557.494	565.5144	567.8197	569.4805	569.177	559.39	560.3074
-0.16	550.5712	555.6651	562.0794	560.4978	562.4995	568.8958	574.4177	561.8167	577.989	576.3918	569.7191
-0.17	547.715	555.8172	557.8041	557.784	560.3853	550.3777	568.5683	565.504	563.6931	562.6992	568.5274
-0.18	546.8171	547.6695	554.2373	552.7206	567.4523	568.8822	567.5718	560.5499	569.7673	559.3997	567.9242
-0.19	536.3453	542.5257	548.3789	555.5535	559.8904	556.996	564.0605	571.4836	557.0458	566.6485	569.4074

a\b	3	3.1	3.2	3.3	3.4	3.5	3.6	3.7	3.8	3.9	4
-0.2	506.5759	501.9225	502.5777	499.9129	502.1328	514.5235	499.3039	497.091	505.1612	501.3162	509.6369
-0.21	504.6174	504.6124	504.7601	506.3674	505.6665	504.7403	505.5692	503.7048	502.2431	496.0927	507.9903
-0.22	491.6412	502.9146	493.039	491.543	492.4929	497.5729	496.1472	510.0655	495.6277	504.7936	499.3552
-0.23	500.5101	505.5784	510.9173	500.1722	497.6127	499.8832	501.4564	500.4376	506.5266	505.5292	506.7824
-0.24	491.1251	492.4665	502.6417	501.048	496.3782	493.035	499.5136	498.1363	505.0667	501.279	501.1975
-0.25	493.229	486.5413	500.7184	492.9377	505.7978	495.5821	500.0638	507.8493	494.9542	495.8567	498.3453
-0.26	496.4411	488.1138	493.1945	498.3509	501.2762	489.9631	496.645	488.4037	499.3305	496.1051	506.0926
-0.27	491.1743	497.4738	494.5892	504.1844	494.6368	496.2717	497.2647	496.9993	494.1603	498.959	498.4212
-0.28	490.0558	492.5272	492.1706	482.5291	490.4117	490.0093	490.836	492.9544	492.8309	500.5089	507.3158
-0.29	480.1516	487.6759	486.3877	487.986	496.3321	493.7066	503.3312	490.6083	499.2633	497.3971	493.5246

a\b	4	4.1	4.2	4.3	4.4	4.5	4.6	4.7	4.8	4.9	5
-0.3	562.2711	560.1333	572.1913	573.8319	558.4417	564.9619	574.8895	571.3489	559.1388	567.7822	570.2627
-0.31	556.5336	547.6758	562.7958	556.8653	569.1892	567.6447	564.3182	572.7176	562.6113	575.7552	557.034
-0.32	565.9787	563.6235	561.6252	552.2063	565.4149	562.3603	565.2108	569.9771	570.1492	556.6738	576.9373
-0.33	558.1402	566.6837	566.1203	559.2991	566.4765	572.9223	566.3339	560.2947	566.9445	560.5397	565.1538
-0.34	549.6286	557.7474	559.1332	554.9696	564.9343	558.3851	560.8831	560.1149	563.228	557.7723	566.7169
-0.35	556.0174	559.3435	546.1252	561.126	562.5968	567.5678	563.9844	561.9805	565.2076	571.1766	564.8133
-0.36	563.1831	558.2149	541.2954	559.9209	561.2748	569.7141	563.3001	572.8615	556.0283	556.6986	563.9661
-0.37	561.7822	554.5341	552.2743	570.8942	569.8	551.906	557.0437	568.7604	549.2566	548.8585	570.9175
-0.38	553.3381	551.0676	550.1977	559.736	550.1014	556.8828	551.8079	563.0973	561.3062	568.488	558.4879
-0.39	555.1326	544.5268	553.1958	565.2869	555.4363	565.7784	558.12	562.9386	562.5978	573.7495	570.1055
